# Infrared saturation and phases of gauge theories with BRST symmetry

**DOI:** 10.1140/epjc/s10052-014-2881-8

**Published:** 2014-05-20

**Authors:** Valentin Mader, Martin Schaden, Daniel Zwanziger, Reinhard Alkofer

**Affiliations:** 1 Institut für Physik, Karl-Franzens-Universität Graz, 8010 Graz, Austria; 2 Department of Physics, Rutgers, The State University of New Jersey, 101 Warren Street, Newark, NJ 07102 USA; 3 Physics Department, New York University, 4 Washington Place, New York, NY 10003 USA

## Abstract

We investigate the infrared limit of the quantum equation of motion of the gauge boson propagator in various gauges and models with a BRST symmetry. We find that the saturation of this equation at low momenta distinguishes between the Coulomb, Higgs and confining phase of the gauge theory. The Coulomb phase is characterized by a massless gauge boson. Physical states contribute to the saturation of the transverse equation of motion of the gauge boson at low momenta in the Higgs phase, while the saturation is entirely due to unphysical degrees of freedom in the confining phase. This corollary to the Kugo–Ojima confinement criterion in linear covariant gauges also is sufficient for confinement in general covariant gauges with BRST and anti-BRST symmetry, maximal Abelian gauges with an equivariant BRST symmetry, non-covariant Coulomb gauge and in the Gribov–Zwanziger theory.

## Introduction

In their seminal work, [1], Kugo and Ojima develop the covariant operator formalism for gauge theories in linear covariant gauge. On the assumption of an unbroken BRST symmetry, they construct the physical Hilbert space of the theory and formulate a criterion for color confinement. The Hilbert space of a gauge theory is defined by the cohomology of $s$, the nilpotent generator of BRST transformations,

1 $$\begin{aligned} {\mathcal H}_\mathrm{phys} = \overline{{{\mathrm{Ker}}}{s}/{{\mathrm{Im}}}{s}}. \end{aligned}$$

The confining phase of a gauge theory according to [1] is characterized by an unbroken global color symmetry *and* the absence of massless gauge bosons. In contrast to criteria based on the behavior of the Wilson loop, the Kugo–Ojima confinement criterion does not depend on the matter content of the theory.

Kugo and Ojima consider the conserved color current operator $J_\mu ^a$ of Yang–Mills theory in linear covariant gauge,

2 $$\begin{aligned} J_\mu ^a = -\partial _\nu F_{\nu \mu }^a + i s (D_\mu \bar{c})^a . \end{aligned}$$

$J_\mu ^a$ is related to the canonical Noether current $j_\mu ^a$ by the quantum equation of motion (QEoM) of the gluon,

3 $$\begin{aligned} j_\mu ^a=J_\mu ^a -\frac{\delta S}{\delta A^a_\mu }, \end{aligned}$$

where $S$ is the gauge-fixed action. According to Kugo and Ojima, color confinement is realized if neither of the two currents

4 $$\begin{aligned} \mathcal {G}_\mu ^a = -\partial _\nu F_{\nu \mu }^a\qquad \text {and}\qquad \mathcal {N}_\mu ^a = i s (D_\mu \bar{c})^a \end{aligned}$$

create massless excitations. The corresponding charge operators

5 $$\begin{aligned} \hbox {G}^a = \int \!\hbox {d}^3x\,\mathcal {G}_0^a \qquad \text {and}\qquad \hbox {N}^a = \int \!\hbox {d}^3x\,\mathcal {N}_0^a \end{aligned}$$

are then both well defined. The global color charge $Q^a = \hbox {G}^a + \hbox {N}^a$ then also is well defined and vanishes on the physical Hilbert space.

Following [1, 2], we introduce the function $u(p^2)$ by the correlator

6 $$\begin{aligned} i\left\langle (D_\mu c)^a\,(D_\nu \bar{c})^b \right\rangle _\mathcal {FT}= \delta ^{ab}\left( T_{\mu \nu }\,u(p^2) - L_{\mu \nu } \right) , \end{aligned}$$

where $L_{\mu \nu } = p_\mu p_\nu /p^2$ and $T_{\mu \nu } = \delta _{\mu \nu } - L_{\mu \nu }$ are longitudinal and transverse projectors, and $\mathcal {FT}$ means Fourier transform. The BRST-exact charge $N^a$ is well defined only if

7 $$\begin{aligned} u(p^2) \xrightarrow {p^2\rightarrow 0} -1. \end{aligned}$$

Together with the absence of massless vector bosons, Eq. (7) is a *sufficient* condition for color confinement [2]. Here we express the confinement criterion of Kugo and Ojima in terms of the saturation of the gluonic QEoM,

8 $$\begin{aligned} \left\langle A_\mu ^a(x) {\delta S \over \delta A_\nu ^b(y)} \right\rangle = \delta _{\mu \nu } \delta ^{ab} \delta (x-y), \end{aligned}$$

at vanishing momentum. With the classically conserved current of global color symmetry $j_\mu ^a$ of Eq. (3), this equation in linear covariant gauge reads

9 $$\begin{aligned} \delta ^{ab}\delta _{\sigma \mu } = -\left\langle A_\sigma ^a \partial _\nu F_{\nu \mu }^b \right\rangle _\mathcal {FT}-\left\langle A_\sigma ^a \left( j_\mu ^b-is(D_\mu \bar{c})^b\right) \right\rangle _\mathcal {FT}.\nonumber \\ \end{aligned}$$

If $\mathcal {G}_\mu ^a$ does not create a massless vector boson, the first correlation function in Eq. (9) vanishes in the infrared limit $p^2\rightarrow 0$ and the current,

10 $$\begin{aligned} \tilde{j}_\mu ^b=j_\mu ^b-is(D_\mu \bar{c})^b, \end{aligned}$$

must saturate Eq. (9) at vanishing momentum. The current $\tilde{j}_\mu ^b$ is physically equivalent to the classical color current $j_\mu ^b$ because they differ by a BRST-exact term only. Condition (7) for the correlator (6) implies that

11 $$\begin{aligned} i\left\langle A_\sigma ^a\,\ s(D_\mu \bar{c})^b \right\rangle _\mathcal {FT}\xrightarrow {p^2\rightarrow 0} \delta ^{ab}\delta _{\mu \nu } .\end{aligned}$$

Thus: *If the Kugo–Ojima criterion is fulfilled, the gluonic QEoM Eq.* (9) *in linear covariant gauge at vanishing momentum is saturated by BRST-exact states.* Since the Physical Hilbert space does not include BRST-exact states, only unphysical states contribute to the saturation of the gluonic QEoM at vanishing momentum.

We therefore have the following

### **Proposition:**

In the confining phase of a gauge theory, unphysical states created by the color current $\tilde{j}_\mu ^a$ saturate the gluonic QEoM at vanishing momentum.

Since confinement allows only color singlet asymptotic states and any asymptotic state that contributes to the gluonic QEoM at vanishing momentum is in the adjoint color representation, the proposition clearly holds. An adjoint multiplet of physical asymptotic states on the other hand exists in Higgs and Coulomb phases. Thus one can discriminate between the Higgs and confinement phase in linear covariant gauge by whether or not physical states contribute to the matrix element of the (generalized) color current $\tilde{j}_\mu ^b$ at vanishing momentum.

It is another matter to prove that the gluonic QEoM indeed *is* saturated by unphysical states at vanishing momentum. Although we do not show this, we find that BRST-exact states in principle can saturate the gluonic QEoM at vanishing momentum in various gauges with a BRST or equivariant BRST symmetry. In particular, we verify in these gauges that

(i) if the theory does not describe the Coulomb phase, the gluonic QEoM at vanishing momentum is saturated by the matrix element with a current that is physically equivalent to the conserved color current, and
(ii) in non-Abelian gauge theories BRST-exact terms exist that can saturate the gluonic QEoM at low momentum.

Since physical states contribute to the gluonic QEoM at vanishing momentum in Higgs and Coulomb phases, we conclude that saturation of the gluonic QEoM at vanishing momentum by a BRST-exact term of the generalized current is a sufficient condition for confinement, provided that the BRST (or equivariant BRST) symmetry is unbroken.

In this article we identify the generalized current and the unphysical BRST-exact term in the gluonic QEoM in various gauges as well as in the Gribov–Zwanziger theory. The emergence of a similar pattern in all these models supports our proposition.

In a lattice theory with fundamental scalars, the Higgs and confining phases at finite lattice coupling $\beta$ are analytically connected [3–5]. The situation is akin to a vapor–liquid transition below the critical point, and a (first-order) transition does seem to occur at sufficiently large $\beta$. It is therefore not clear whether the two phases remain analytically connected in the continuum limit. Since color charge is screened by a Higgs (or quark) field in the fundamental representation, the asymptotic behavior of the Wilson loop is expected to always be perimeter-like and cannot be used to distinguish between phases. As the liquid–vapor transition below the critical point demonstrates, the absence of an order parameter does not necessarily imply that the free energy is analytic everywhere. Gauge-dependent criteria for Higgs and confining phases give different critical curves *below* the critical $\beta$-value, but are remarkably consistent *above* this critical point [5]. Analogous results have been obtained in a semi-classical continuum analysis of a non-Abelian Higgs model within the Gribov-horizon [6, 7].

However, to unambiguously distinguish between a Higgs and a confining phase, we in this article consider only Yang–Mills theory without fundamental matter. This gauge theory is either in a Coulomb, a Higgs or a confining phase and the asymptotic behavior of the Wilson (or of the ’t Hooft) loop [8] distinguishes between the last two. The investigation of transitions between these phases is beyond the scope of the present article.

We will consider Yang–Mills theory in linear covariant (LCG), generalized linear covariant (GLCG), maximal Abelian (MAG), Coulomb (CG) and minimal Landau (GZ) gauge. Some known results for these gauges are summarized below.

The best investigated Linear Covariant Gauge (LCG) is Landau gauge. Another corollary of the Kugo–Ojima confinement criterion [2] in this gauge relates the infrared limit of the ghost dressing function $G(p^2)$ to $u(p^2)$, $\lim _{p^2\rightarrow 0}G(p^2)^{-1} = 1+ \lim _{p^2\rightarrow 0} u(p^2)$. When Eq. (7) is fulfilled, the ghost dressing function diverges in the infrared, and the ghost propagator at vanishing momentum is more singular than a massless pole. An exact infrared analysis of the whole tower of Dyson–Schwinger (DSE) and of Exact Renormalization Group Equations (ERGE) confirms the existence of solutions with this infrared behavior of the ghost propagator and a corresponding infrared suppressed gluon propagator; see for instance Refs. [9–18]. The solutions for which the Kugo–Ojima criterion is fulfilled have a power-like enhancement of the ghost propagator with related infrared exponents of other Green functions determined by an infinite tower of scaling relations [15, 16]. According to numerical studies of Yang–Mills theory in 2d [19–21], the ghost and gluon propagators exhibit this scaling behavior, and, moreover, a strict analytic bound [22] implies that in the latter case the gluon propagator vanishes at zero momentum $D(0) = 0$.

However, in 3d and 4d another one-parameter family of solutions to the DSEs and ERGEs also exists that is best parameterized by the value of $G(0)^{-1}\ne 0$. The ghost propagator of these solutions is only quantitatively enhanced and the gluon propagator is infrared finite [17, 18, 23, 24]. Lattice gauge theory studies in three and four dimensions observe only this infrared finite behavior [25–28] of lattice propagators. But a whole one-parameter family of solutions can also be obtained on the lattice by tuning the lowest eigenvalue of the Faddeev–Popov operator [29] of the gauge fixing. The origin of this multitude of solutions is unresolved and it has been suggested [25] that the value of $G(0)^{-1}$ could be considered an additional gauge parameter. Numerical solutions over the whole momentum range are only available for truncated DSEs and ERGEs.

Within the error of the employed approximation and/or truncation, both types of solutions lead to very similar phenomenology [30] and confine static quarks [31]. Unbroken BRST symmetry is essential for the Kugo–Ojima confinement criterion. Without recourse to a preserved nilpotent symmetry it is difficult to identify the unphysical sector of such truncated models. However, our proposition that confinement is a Higgs mechanism in the unphysical sector of the theory can be formulated in the absence of a nilpotent symmetry and may hold in all these scenarios.

In the minimal Coulomb gauge, the dressing function of the ghost propagator of Yang–Mills theory is more divergent than a simple massless pole, effectively leading to a confining color-Coulomb potential [32]. The infrared divergence of the instantaneous ghost propagator in this gauge has been verified by other calculations in the continuum [33–35] and on the lattice [36]. For a thorough discussion of the current status see [37].

The dual superconductor picture of the QCD vacuum [39, 40] is the motivation for considering Yang–Mills in maximal Abelian gauge (MAG), [41–45]. This gauge discriminates between the Cartan subalgebra and the coset space of the gauge group and the partial gauge fixing breaks the local $\hbox {SU}(N)$ invariance down to the Cartan subgroup ${U(1)}^{N-1}$. The hypothesis of Abelian dominance [46] states that the Cartan gluons dominate long-range interactions. This has been observed in lattice simulations [47, 48] and is also corroborated by an infrared analysis of the functional equations [49, 50]. Furthermore, the Cartan gluons also dominate at large momenta [51, 52]. A detailed understanding of the relation between the infrared behavior of Green’s functions and confinement nevertheless is lacking in the MAG. It is of interest that a renormalization group analysis of interpolating gauges found that Abelian gauges form an invariant subspace that is not analytically connected to linear covariant gauges [53]. This explains why a literal interpretation of the Kugo–Ojima confinement criterion fails for this class of gauges [54]. Our proposition that unphysical states saturate the gluonic QEoM at vanishing momentum can nevertheless be realized. Contrary to Abelian gauge theories, the Abelian current of non-Abelian gauge theories includes an operator that only creates unphysical states that could saturate the gluonic QEoM.

The Gribov–Zwanziger framework [55–59] restricts the path integral to the first Gribov region of LCG and Coulomb gauge. Even though it drastically changes the gauge-fixed action and breaks BRST symmetry spontaneously, [60, 61], it does not change the form of the DSEs in Landau gauge [32], and the infrared exponent of the scaling solution is that of ordinary Landau gauge [62, 63]. We find that the color current of this model includes a BRST-exact contribution. The latter in fact saturates the gluonic QEoM at vanishing momentum already at tree level. However, it is difficult to verify that this BRST-exact operator only creates unphysical states since the BRST symmetry of this model is spontaneously broken.

Yang–Mills theory is expected to confine in all these gauges, and one hopes that the underlying mechanism can be characterized in a gauge-invariant fashion. Although the dynamics may be different, our proposition—concerning saturation of the gluonic QEoM at vanishing momentum by unphysical states—can be realized in all of them. Other similarities include that the dielectric function of the QCD vacuum appears to be related to the divergent dressing function of the ghost propagator in Coulomb gauge [64], and that in the Landau gauge the Kugo–Ojima criterion is related to the Gribov–Zwanziger scenario [65].

The present article is organized as follows: In Sect. 2, we examine the gluonic QEoM in Abelian gauge theory in linear covariant gauges, and we review the arguments that distinguish between Coulomb and Higgs phases in this specific case. In Sect. 2.1, we examine the Abelian Coulomb phase in greater detail, and in Sect. 2.2 we explicitly verify the implications of a spontaneously broken ${U(1)}$-symmetry in the t’ Hooft gauge. In Sect. 3.1 the Kugo–Ojima confinement criterion for LCG is reviewed. We then proceed to generalize and adapt this criterion to other gauges: to generalized covariant gauges in Sect. 3.2, to covariant but non-linear MAG in Sect. 3.3, and to the non-covariant Coulomb gauge in Sect. 3.4. In all these gauges a Kugo–Ojima-like confinement criterion is formulated. In Sect. 3.5 we examine signatures of confinement in the Gribov–Zwanziger (GZ) theory. Section 4 summarizes our results. Conventions and some technical details are deferred to three appendices.

## Abelian gauge theories

In Abelian gauge theories one can, of course, dispense with a BRST construction of observables [66, 67]. However, identifying the physical sector by a BRST symmetry is readily extended to non-Abelian gauge theories, and to non-canonical quantization.

To this end we consider Abelian gauge theories in general linear covariant gauges,

12 $$\begin{aligned} \mathcal L_{U(1)}&= \mathcal L_A+ \mathcal L_M+s\left( \bar{c}\left( \frac{\xi }{2} b -i\partial _\mu A_\mu +i \gamma (\phi ,\dots )\right) \right) \nonumber \\&=\mathcal L_A+ \mathcal L_M+\frac{\xi }{2} b^2 -ib\partial _\mu A_\mu +i b\gamma (\phi ,\dots ) \nonumber \\&\quad +i\bar{c}\partial ^2 c-i\bar{c} s\gamma (\phi ,\dots ). \end{aligned}$$

Here $\xi$ is a gauge parameter and $b,c,$ and $\bar{c}$ are the Nakanishi–Lautrup (NL) and (anticommuting) ghost and antighost fields. The local function $\gamma (\dots )$ of canonical dimension $2$ and vanishing ghost number is a polynomial of bosonic matter fields $\phi$ that does not depend on the gauge connection $A_\mu$ or the NL field $b$. The matter part, $\mathcal L_M$, is invariant under ${U(1)}$-gauge transformations and may include covariantly coupled charged fermions and bosons.

The variation $s$ generates the nilpotent BRST symmetry [68, 69] of $\mathcal L_{U(1)}$ (12),

13 $$\begin{aligned} s A_\mu =\partial _\mu c, \quad s c =0, \quad s \bar{c} =b, \quad s b&=0\, . \end{aligned}$$

Under the BRST variation $s$, charged matter fields vary by an infinitesimal ${U(1)}$-gauge transformation with the ghost field $c(x)$ as variation. The longitudinal gauge field, ghost $c$, antighost $\bar{c}$ and NL field $b$ form the elementary BRST quartet [1, 70]. We note that one can define an anti-BRST variation in this Abelian setting by

14 $$\begin{aligned} \bar{s} A_\mu =\partial _\mu \bar{c}, \quad \bar{s} \bar{c} =0, \quad \bar{s} c =- b, \quad \bar{s} b =0, \end{aligned}$$

with an obvious extension to matter fields. The generators of BRST and of anti-BRST transformations are nilpotent and anticommute, $s^2=\bar{s}^2=\left\{ s, \bar{s} \right\} =s \bar{s}+\bar{s} s=0$. The conserved BRST and anti-BRST charges corresponding to the transformations in Eqs. (13) and (14) in the Abelian case may be represented by the functional derivative operators,

15a $$\begin{aligned} Q_\mathrm{BRST}&=\int \hbox {d}^4x\left( -c(x)\partial _\mu \frac{\delta }{\delta A_\mu (x)}+ b(x)\frac{\delta }{\delta \bar{c}(x)}\right. \nonumber \\&\quad \left. +\sum _ M(s \phi _M(x)) \frac{\delta }{\delta \phi _M(x)}\right) \end{aligned}$$

15b $$\begin{aligned} \bar{Q}_\mathrm{BRST}&=\int \hbox {d}^4x \left( -\bar{c}(x)\partial _\mu \frac{\delta }{\delta A_\mu (x)}- b(x)\frac{\delta }{\delta c(x)}\right. \nonumber \\&\quad \left. +\sum _ M(\bar{s} \phi _M(x)) \frac{\delta }{\delta \phi _M(x)}\right) \end{aligned}$$

where the sums run over all matter fields $\phi _M(x)$. The ghost number,

16 $$\begin{aligned} \Pi =\int \hbox {d}^4x\left( c(x)\frac{\delta }{\delta c(x)}- \bar{c}(x)\frac{\delta }{\delta \bar{c}(x)}\right) , \end{aligned}$$

also is conserved.

The nilpotent BRST symmetry allows one to define the subset $\mathfrak {P}$ of physical operators by the cohomology [68],

17 $$\begin{aligned} \mathfrak {P}&=\{\text {physical operators}\}\nonumber \\&=\{\mathcal {O};\ [Q_\mathrm{BRST},\mathcal {O}]=0\ \text {and}\ [\mathcal {O},\Pi ]=0\} \nonumber \\&\quad / \{[Q_\mathrm{BRST},\mathcal {O}];\ [\mathcal {O},\Pi ]=\mathcal {O}\}. \end{aligned}$$

Using a canonical construction, it was shown [1] that negative-norm states associated with asymptotic BRST quartets are unphysical. The elementary quartet thus is not observable. Transversely polarized photons, on the other hand, are physical. Instead of constructing the physical asymptotic Hilbert space directly, we prefer to define the space of physical operators, which is a slightly more flexible point of view that can be extended to spaces other than four-dimensional Minkowski spacetime. With a BRST invariant ground state, the two approaches are equivalent in Minkowski space.

Since matter transforms covariantly under ${U(1)}$-gauge transformations, $A_\mu \rightarrow A_\mu +\partial _\mu \theta$, and $\mathcal L_M$ is an invariant, the conserved global ${U(1)}$-current, $j_\mu ^{U(1)}$, is obtained from the matter part of the action alone,

18 $$\begin{aligned} {j_\mu }^{{U(1)}}(x)=-\frac{\delta \mathcal {S}_M}{\delta A_\mu (x)}, \end{aligned}$$

with $$\mathcal {S}_M=\int \hbox {d}^4x \mathcal L_M$$.

The QEoM of the Abelian gauge boson propagator in linear covariant gauges reads

19 $$\begin{aligned} \delta _{\sigma \mu }\delta (x-y)&= \left\langle A_\sigma (y)\,\frac{\delta S_{U(1)}}{\delta A_\mu (x)} \right\rangle \nonumber \\&= -\left\langle A_\sigma (y)\, \partial _\nu F_{\nu \mu }(x) \right\rangle -\left\langle A_\sigma (y)\, {j_\mu }^{{U(1)}}(x) \right\rangle \nonumber \\&\quad + i\left\langle A_\sigma (y)\, s \partial _\mu \bar{c}(x) \right\rangle . \end{aligned}$$

It depends on the classically conserved current ${j_\mu }^{{U(1)}}(x)$ of Eq. (18) and holds for renormalized as well as for bare fields. The last, longitudinal, term on the r.h.s. in Eq. (19) arises from the linear covariant gauge fixing in Eq. (12) and is BRST-exact. It has no physically observable effects and may be included in the definition of the current ${\tilde{j}_\mu }^{U(1)}(x)={j_\mu }^{{U(1)}}(x)-i s \partial _\mu \bar{c}$. In close analogy to the non-Abelian case discussed below one can use Eq. (14) to write, $s \partial _\mu \bar{c} = s \bar{s} A_\mu$.

The first term on the r.h.s. of Eq. (19) is transverse due to the antisymmetry of the field strength tensor. Fourier transformation (denoted by $\left\langle \dots \right\rangle _\mathcal {FT}$) of Eq. (19) and longitudinal ($L_{\mu \nu } = {p_\mu p_\nu }/{p^2}$) and transverse ($T_{\mu \nu } = \delta _{\mu \nu } - L_{\mu \nu }$) projection give the identities,

20a $$\begin{aligned} L_{\sigma \mu }&=\left\langle A_\sigma (y)\, i \partial _\mu s\bar{c}(x) \right\rangle _\mathcal {FT}- L_{\mu \rho }\left\langle A_\sigma (y)\, {j_\rho }^{{U(1)}}(x) \right\rangle _\mathcal {FT},\end{aligned}$$

20b $$\begin{aligned} T_{\sigma \mu }&= -\left\langle A_\sigma (y)\, \partial _\nu F_{\nu \mu } \right\rangle _\mathcal {FT}-T_{\mu \rho }\left\langle A_\sigma (y)\,{j_\rho }^{{U(1)}}(x) \right\rangle _\mathcal {FT}. \end{aligned}$$

Using the equation of motion of the NL field, Eq. (20a) yields the Ward identity for the longitudinal photon propagator,

21 $$\begin{aligned} \xi \, p_\sigma&= p^2 p_\nu \,\left\langle A_\sigma (y)\,A_\nu (x) \right\rangle _\mathcal {FT}-ip^2\left\langle A_\sigma (y)\, \gamma (x) \right\rangle _\mathcal {FT}\nonumber \\&- i\xi \left\langle A_\sigma (y)\,\partial _\nu {j_\nu }^{{U(1)}}(x) \right\rangle _\mathcal {FT}, \end{aligned}$$

where $\gamma (x)=\gamma (\phi (x),\dots )$ is the local function of the fields in the BRST-exact term of Eq. (12).

The correlator of the gauge field with the divergence of the field strength tensor is transverse and is described by a Lorentz invariant function $f(p^2)$,

22 $$\begin{aligned} T_{\sigma \mu } f(p^2):=-\left\langle A_\sigma (y)\, \partial _\nu F_{\nu \mu }(x) \right\rangle _\mathcal {FT}, \end{aligned}$$

which for $p^2\rightarrow 0$ determines the phase of the model. $f(0)\ne 0$ implies a pole at $p^2=0$ due to a massless transverse vector boson in the correlator

23 $$\begin{aligned} \left\langle A_\sigma (y)\, F_{\nu \mu }(x) \right\rangle _\mathcal {FT}=-i (\delta _{\sigma \mu } p_\nu - \delta _{\sigma \nu }p_\mu ) \frac{f(p^2)}{p^2}. \end{aligned}$$

A model with $f(0)\ne 0$ thus has a massless photon and describes a Coulomb phase. The Abelian nature of the field strength is not essential for inferring a massless pole in Eq. (23). One merely exploits the Poincaré invariance and the antisymmetry of the curvature $F_{\mu \nu }$.

In the Abelian case, $f(p^2)$ determines the transverse part of the vector boson propagator,

24 $$\begin{aligned} T_{\mu \nu } \, \left\langle A_\sigma (y)\,A_\nu (x) \right\rangle _\mathcal {FT}=\frac{f(p^2)}{p^2} T_{\sigma \mu }, \end{aligned}$$

and the photon is massless only if $f(0)>0$. Insertion of the definition, Eq. (22), into Eq. (20b) shows that $f(p^2)$ also completely determines the *transverse* current matrix element,

25 $$\begin{aligned} T_{\mu \nu }\left\langle A_\sigma (y)\,{j_\nu }^{{U(1)}}(x) \right\rangle _\mathcal {FT}=(f(p^2)-1)T_{\sigma \mu }. \end{aligned}$$

If the current saturates Eq. (20b) in the infrared,

26 $$\begin{aligned} T_{\mu \nu }\left\langle A_\sigma (y)\,{j_\nu }^{{U(1)}}(x) \right\rangle _\mathcal {FT}\mathop {\xrightarrow {}}\limits ^{p^2\rightarrow 0}-T_{\sigma \mu }, \end{aligned}$$

the vector boson is short ranged, and $f(0)=0$.

These relations hold for any Abelian gauge theory in linear covariant gauges. $f(0)\ne 0$ characterizes a model describing a Coulomb phase with a massless photon. We next examine Abelian gauge theories in the Coulomb and Higgs phase in more detail. In Sect. 3.3 we consider an Abelian gauge theory that confines color charge and investigate possible signatures of this phase.

### The Coulomb phase

The Coulomb phase is characterized by an unbroken Abelian gauge symmetry *and* the failure of Eq. (26), i.e., failure of the current contribution to saturate Eq. (20b) in the infrared. Since the Abelian gauge symmetry is unbroken, the correlation function $\langle A_\sigma (y)\,{j_\nu }^{{U(1)}}(x)\rangle$ is transverse in any covariant gauge. Equation (20a) and the Ward identity Eq. (21) have the form

27 $$\begin{aligned} L_{\sigma \mu }&= p_\mu \left\langle A_\sigma (y)\, b(x) \right\rangle _\mathcal {FT}\nonumber \\&\text {and}\ \ \xi \, \frac{p_\sigma }{p^2}= p_\nu \,\left\langle A_\sigma (y)\,A_\nu (x) \right\rangle _\mathcal {FT}. \end{aligned}$$

The elementary quartet is free and massless:

28 $$\begin{aligned} \left\langle A_\mu (y)\,b(x) \right\rangle _\mathcal {FT}=\left\langle \partial _\mu c(y)\, \bar{c}(x) \right\rangle _\mathcal {FT}=\frac{p_\mu }{p^2}. \end{aligned}$$

From Eq. (24) and Eq. (27) the photon propagator is given by

29 $$\begin{aligned} \left\langle A_\mu (y)\,A_\nu (x) \right\rangle _\mathcal {FT}= \frac{f(p^2)}{p^2} T_{\mu \nu }+ \frac{\xi }{p^2} L_{\mu \nu }. \end{aligned}$$

The photon is massless with $f(0)>0$, because Eq. (26) does not hold.

It is interesting to note that in the canonical formalism $f(0)\ne 0$ implies that the electromagnetic charge operator,

30 $$\begin{aligned} Q=\int \hbox {d}^3x {j_0}^{{U(1)}}(x), \end{aligned}$$

is not well defined. Up to terms proportional to the photon equation of motion, this charge is equivalent to

31 $$\begin{aligned} Q\equiv \tilde{Q}&= \int \hbox {d}^3x (i\partial _0 b(x)-\partial _\nu F_{\nu 0}(x)) \nonumber \\&= \int \hbox {d}^3x i\partial _0 b(x)+\int _{S_\infty } d{\mathbf \sigma }_i F_{0i}=\mathcal {N}+\mathcal G. \end{aligned}$$

Due to the antisymmetry of the field strength tensor, $F_{\nu \mu }=-F_{\mu \nu }$, the current $-\partial _\nu F_{\nu \mu }(x)$ and corresponding charge $\mathcal G$ are themselves conserved. Furthermore, the equal time commutator of $\mathcal G$ with any *local* physical operator $\Phi (x)\in \mathfrak {P}$ vanishes,

32 $$\begin{aligned}{}[\Phi (x),\mathcal G]=0\ \text { for all } local\ \Phi (x)\in \mathfrak {P}, \end{aligned}$$

because causality requires operators with spatial separation to commute. One thus has

33 $$\begin{aligned} \left[ Q, \Phi (x) \right]&\equiv \left[ \mathcal {N}+\mathcal G, \Phi (x) \right] =\left[ \left\{ \mathcal C, Q_\mathrm{BRST} \right\} , \Phi (x) \right] \nonumber \\&=\left\{ \mathcal C, \left[ Q_\mathrm{BRST}, \Phi (x) \right] \right\} +\left\{ Q_\mathrm{BRST}, \left[ \mathcal C, \Phi (x) \right] \right\} \nonumber \\&=\left\{ Q_\mathrm{BRST}, \left[ \mathcal C, \Phi (x) \right] \right\} \end{aligned}$$

for any *local* $\Phi (x)\in \mathfrak {P}$. Here $\mathcal C=i\int \hbox {d}^3 x \partial _0 \bar{c} =\int \hbox {d}^3 x \pi _c(x)$ is the canonical conjugate of the ghost operator at vanishing momentum and $\mathcal {N}= \left\{ Q_\mathrm{BRST}, {\mathcal C} \right\}$. In deriving Eq. (33) one uses causality, the Jacobi identity and that $\mathcal {N}$ is BRST exact. All *local physical* operators $\Phi (x)\in \mathfrak {P}$ thus are uncharged and physical operators creating charged particles like the electron necessarily are not local. (NB: To compare with non-Abelian gauge theories, note that $Q_\mathrm{BRST}$ and ${\mathcal C}$ may be replaced by $\bar{Q}_\mathrm{BRST}$ and $\bar{\mathcal C}=\int \hbox {d}^3 x \pi _{\bar{c}}(x)$ in Eq. (33).) One *can* construct non-local charged physical states in QED because infrared photon states are almost degenerate with the ground state [71–74]. The massless vector boson of the Coulomb phase thus prevents one from concluding from Eq. (33) that all physical states are uncharged.

### The Abelian Higgs phase 

A “spontaneously broken” Abelian gauge theory in the Higgs phase satisfies Eq. (19) differently. From the general discussion one expects that $f(0)=0$, the vector boson is massive and the current saturates Eq. (20b) at low momenta, *i.e.*, Eq. (26) holds. In the Higgs phase one also expects (unphysical) massless excitations. We explicitly verify this scenario in QED with charged scalar matter whose self-interactions are described by a Higgs potential with quartic coupling $\lambda$ and a negative quadratic term proportional to $-4\lambda v^2$,

34a $$\begin{aligned} \mathcal L_M^\text {Higgs}&= \frac{1}{2} (D_\mu \Phi )^* (D_\mu \Phi ) + \lambda \left( \left| \Phi \right| ^2 - v^2\right) ^2 \end{aligned}$$

34b $$\begin{aligned}&= \frac{1}{2} \left( (\partial _\mu \phi _+)^2 + (\partial _\mu \phi _-)^2 \right) + \frac{m^2}{2}\,A_\mu ^2 + m\, \phi _- \partial _\mu A_\mu \nonumber \\&\quad + g \, A_\mu \left( \phi _-\partial _\mu \phi _+-\phi _+\partial _\mu \phi _-\right) + gm \, A_\mu ^2 \phi _+\\&\quad + \frac{g^2}{2}\,A_\mu ^2 \left( \phi _+^2 + \phi _-^2 \right) + \lambda \left( \phi _+^2 + \phi _-^2 + 2 \phi _+v \right) ^2 . \nonumber \end{aligned}$$

In the Higgs phase with $v>0$ we parameterize the fields by $\Phi = \phi + v ,\, \phi _+= {\frac{1}{2}} (\phi ^*+\phi ) ,\, \phi _-={\frac{i}{2}}( \phi ^*-\phi )$. The tree-level photon mass is $m = gv$ and that of the Higgs field $\phi _+$ is $m^2_H=8\lambda v^2$. $\phi _-$ is massless and couples to the longitudinal photon. The model is invariant under local gauge transformations $\delta A_\mu = \partial _\mu \theta ,\, \delta \Phi = i g \theta \Phi ,\, \delta \phi _+= - g \theta \phi _-,\, \delta \phi _-= g \theta \left( \phi _++ v \right)$. Replacing $\theta (x)$ by the anticommuting ghost field one arrives at the BRST variations

35a $$\begin{aligned} s A_\mu&= \partial _\mu c ,\qquad sc = 0 ,\qquad s \bar{c} = b ,\qquad sb = 0,\end{aligned}$$

35b $$\begin{aligned} s\phi _+&= -gc \,\phi _-,\qquad s \phi _-= g c \left( \phi _++ v \right) . \end{aligned}$$

A convenient gauge that eliminates the bilinear coupling of $\phi _-$ to the longitudinal photon is given by the BRST-exact linear covariant gauge-fixing term [75],

36 $$\begin{aligned} \mathcal L_\mathrm{GF}^\text {'t Hooft}&= s\left( \bar{c} \left( -i \partial _\mu A_\mu + \frac{\xi }{2} b + i\xi m \phi _-\right) \right) \nonumber \\&= \frac{\xi }{2} b'^2 - i b' \partial _\mu A_\mu - m \, \phi _-\partial _\mu A_\mu + \frac{\xi m^2}{2}\,\phi _-^2\nonumber \\&\quad + i \bar{c} \left( \partial ^2 - gm\xi \phi _+- m^2 \xi \right) c, \end{aligned}$$

where in the second expression the NL field has been shifted: $b = b' -i m \phi _-$. The classical Lagrangian of the Abelian Higgs model in linear covariant ’t Hooft gauge is

37 $$\begin{aligned} \mathcal L^\text {Higgs} = \mathcal L_A+\mathcal L_M^\text {Higgs} + \mathcal L_\mathrm{GF}^\text {'t Hooft}. \end{aligned}$$

Note that the BRST-exact term $\mathcal L_\mathrm{GF}^\text {'t Hooft}$ of Eq. (37) explicitly breaks not only local but also global ${U(1)}$-symmetry.

The QEoM of the photon propagator is given by Eq. (19) with the gauge-invariant and classically conserved matter current

38 $$\begin{aligned} {j_\mu }^{{U(1)}}&= \delta \Phi \,\frac{\delta \mathcal L_M^\text {Higgs}}{\delta \partial _\mu \Phi } + \delta \Phi ^* \,\frac{\delta \mathcal L_M^\text {Higgs}}{\delta \partial _\mu \Phi ^*} \nonumber \\&= g \left( \phi _+\partial _\mu \phi _--\phi _-\partial _\mu \phi _+\right) + m \partial _\mu \phi _-\nonumber \\&\qquad - A_\mu \left( g^2( \phi _+^2 + \phi _-^2)+m^2 +2 mg \phi _+\right) . \end{aligned}$$

The current is BRST invariant, and its divergence is unphysical because the global gauge invariance of the model is broken by BRST exact terms only. To leading order in the loop expansion one has

39 $$\begin{aligned} s {j_\mu }^{{U(1)}}\approx s(m\partial _\mu \phi _-- m^2 A_\mu )=gm\partial _\mu (c\phi _+) \approx 0. \end{aligned}$$

In fact, the divergence $\partial _\mu {j_\mu }^{{U(1)}}$ is BRST exact up to equations of motion. In leading approximation

40 $$\begin{aligned}&\partial _\mu {j_\mu }^{{U(1)}}\approx m\partial ^2\phi _--m^2 \partial _\mu A_\mu \nonumber \\&\quad \equiv m^2(\xi m\phi _-- \partial _\mu A_\mu ) \equiv i m^2\xi b=i m^2\xi s\bar{c}, \end{aligned}$$

where the tree-level QEoM of $\phi _-$ and of the NL field $b$ was used to obtain the intermediate expressions.

In the broken phase, the current contribution to Eq. (20a) does not vanish and in fact saturates it at low momenta. This is the signature of a “spontaneously broken” gauge theory. Since the divergence of the current is BRST exact up to equations of motion, it does not create physically observable Goldstone bosons and the gauge theory is in a Higgs phase. In ’t Hooft gauges the mass of the unphysical scalar particle created by ${j_\mu }^{{U(1)}}$ depends on the gauge parameter $\xi$ and vanishes for $\xi =0$ only.

With $\gamma (x)=\xi m \phi _-(x)$, the Ward identity of Eq. (21) to leading order asserts,

41 $$\begin{aligned} \xi \, p_\sigma&= p^2 p_\nu \left\langle A_\sigma (y) A_\nu (x) \right\rangle _\mathcal {FT}+i\xi m\left\langle A_\sigma (y) \partial ^2\phi _-(x) \right\rangle _\mathcal {FT}\nonumber \\&\quad - i\xi \left\langle A_\sigma (y)\,\partial _\nu {j_\nu }^{{U(1)}}(x) \right\rangle _\mathcal {FT}\nonumber \\&\approx p^2 p_\nu \left\langle A_\sigma (y) A_\nu (x) \right\rangle _\mathcal {FT}+i\xi m \left\langle A_\sigma (y) \partial ^2\phi _-(x) \right\rangle _\mathcal {FT}\nonumber \\&\quad -i\xi \left\langle A_\sigma (y)\,\partial _\nu (m\partial _\nu \phi _--m^2 A_\nu )(x) \right\rangle _\mathcal {FT}\nonumber \\&= (p^2+\xi m^2) p_\nu \,\left\langle A_\sigma (y)\,A_\nu (x) \right\rangle _\mathcal {FT}, \end{aligned}$$

Note that $\phi _-$ does not contribute to the Ward identity at tree level. This cancelation of mixing terms is a feature of ’t Hooft gauges.

However, the current matrix element in Eq. (41) saturates Eq. (20a) for $p^2\rightarrow 0$ whereas it vanishes in the Coulomb phase in this limit. This is not a gauge artifact and for $\xi \ne 0$ does not depend on the gauge parameter.

Equation (41) gives the tree-level longitudinal propagator in the Higgs phase:

42 $$\begin{aligned}&p_\nu \,\left\langle A_\sigma (y)\,A_\nu (x) \right\rangle _\mathcal {FT}\approx \frac{\xi p_\sigma }{p^2 + \xi m^2} \nonumber \\&\quad =\frac{p_\sigma }{m^2}-\frac{p^2p_\sigma }{m^2(p^2+\xi m^2)}, \end{aligned}$$

which may be directly verified from the quadratic terms of the action Eq. (37). In the last expression of Eq. (42), the $\xi$-independent term at $p^2=0$ arises from the current matrix element. The second term is the $\xi$-dependent negative-norm contribution of $\left\langle A_\sigma (y)\, i\partial _\mu s \bar{c}(x) \right\rangle \approx \frac{p^2}{p^2+\xi m^2} L_{\sigma \mu }$. It is one of the correlators of the elementary BRST quartet and for $\xi m^2\ne 0$ vanishes as $p^2\rightarrow 0$, leaving the current to saturate the Eq. (20a) in the Higgs phase.

In the Higgs phase the tree-level approximation to the function $f(p^2)$ defined by Eq. (22) is

43 $$\begin{aligned} f(p^2) \approx \frac{p^2}{p^2 + m^2} . \end{aligned}$$

Since $f(0)=0$ the transverse vector boson is short ranged in this phase,

44 $$\begin{aligned} T_{\mu \nu } \left\langle A_{\sigma }(y)\,A_\nu (x) \right\rangle _\mathcal {FT}\approx \frac{1}{p^2 + m^2}T_{\sigma \mu } . \end{aligned}$$

The current of Eq. (38) thus saturates Eq. (20b) at low momenta, for any value of the gauge parameter $\xi$ and Eq. (26) holds in the Higgs phase. Note that the physical Higgs particle and vector boson in this model are not charged.

These examples illustrate (at tree level) the characteristics that distinguish the unbroken Coulomb and “spontaneously broken” Higgs phases of Abelian gauge theories. If the current contribution saturates the transverse *and* the longitudinal QEoM of the photon propagator at low momenta, the model describes a “spontaneously broken” Higgs phase. If the current contribution fails to saturate the transverse equation at low momenta, the Abelian gauge theory describes a Coulomb phase with a massless vector particle. The (conserved) transverse part of the Abelian current in our examples is BRST invariant and does not include BRST exact terms. At vanishing momentum it apparently creates only physical particles. This will change when we consider Abelian gauge theories that confine color charge in Sect. 3.3.

First however, let us revisit non-Abelian gauge theories in Linear Covariant Gauges (LCG) for which Kugo and Ojima originally formulated their confinement criterion.

## Non-Abelian gauge theories

### The Kugo–Ojima confinement criterion in linear covariant gauges (LCG)

While the photon is massless and atoms are readily ionized, gluons are only of short range and no hadron has been color-ionized. This confinement of color charge is one of the most prominent features of unbroken non-Abelian gauge theories. One expects the confinement of color and the absence of massless gluons to manifest themselves in solutions to the QEoM of the vector boson propagator. We here give a short review of Kugo and Ojima’s analysis [1] of unbroken non-Abelian gauge theories in the linear covariant gauge (LCG).

Yang–Mills theory in LCG is defined by the Lagrangian

45 $$\begin{aligned} \mathcal L_\mathrm{LCG}&= \mathcal L_\mathrm{YM}+ s \left( \bar{c}^a\left( \frac{\xi }{2} b^a-i \partial _\mu A^a_\mu \right) \right) \nonumber \\&= \mathcal L_\mathrm{YM}+\frac{\xi }{2} b^2 - i b^a \partial _\mu A_\mu ^a - i \partial _\mu \bar{c}^a (D_\mu c)^a . \end{aligned}$$

The nilpotent BRST transformation in the non-Abelian case is given by

46 $$\begin{aligned} s A_\mu ^a&= (D_\mu c)^a, \quad sc^a = -\frac{1}{2} \left( c\! \times \! c \right) ^{a} , \nonumber \\ s \bar{c} ^a&= b^a, \quad sb^a = 0, \end{aligned}$$

and is readily extended to covariantly coupled matter. The Lagrangian (45) is also invariant under an anti-BRST transformation generated by $\bar{s}$,

47 $$\begin{aligned} \bar{s} A_\mu ^a&= (D_\mu \bar{c})^a, \quad \bar{s}\bar{c}^a = -\frac{1}{2} \left( \bar{c}\! \times \! \bar{c} \right) ^{a} ,\nonumber \\ \bar{s} c ^a&= - b^a - \left( \bar{c}\! \times \! c \right) ^{a} , \quad \bar{s} b^a =\left( b\! \times \! \bar{c} \right) ^{a} . \end{aligned}$$

It is in addition invariant under *global* color rotations and preserves ghost number. The BRST and anti-BRST transformations are nilpotent and anticommute, $s^2 = \bar{s}^2 = \left\{ s, \bar{s} \right\} = 0$.

As in the Abelian case, one defines a BRST charge $Q_\mathrm{BRST}$ and anti-BRST charge $\bar{Q}_\mathrm{BRST}$ analogous to Eq. (15). The space $\mathfrak {P}$ of physical operators then is the sector of vanishing ghost number of the BRST cohomology as in Eq. (17). Unphysical states of indefinite norm are associated with BRST quartets. To all orders in perturbation theory these do not contribute to the physical scattering matrix and cannot be created from physical states by physical operators [1, 68]. The longitudinal gauge field, ghost $c$, antighost $\bar{c}$ and NL field $b$ again form the elementary BRST quartet [1, 70]. Contrary to the Abelian case, the transverse gluon of a non-Abelian gauge theory is part a non-perturbative BRST quartet [76–78] and not physical.

In the canonical formulation, the global color symmetry of this theory implies the conserved Noether currents,

48 $$\begin{aligned} j_\mu ^{LCG\,a}&= \left( A_\nu \! \times \! (F_{\nu \mu } + i \delta _{\mu \nu } b) \right) ^{a} \nonumber \\&\quad - i\left( c\! \times \! \partial _\mu \bar{c} \right) ^{a} + i\left( \bar{c}\! \times \! D_\mu c \right) ^{a} . \end{aligned}$$

Up to the gluonic QEoM,

49 $$\begin{aligned} J_\mu ^a = - \partial _\nu F_{\nu \mu }^a + i s \bar{s} A_\mu ^a = - \partial _\nu F_{\nu \mu }^a+i s (D_\mu \bar{c})^a, \end{aligned}$$

is equivalent to $j_\mu ^{LCG\,a}$ and the color charges $\mathcal G^a$ and $\mathcal {N}^a$ in

50 $$\begin{aligned} Q^a=\int \hbox {d}^3x j_0^{LCG\,a}\equiv \mathcal G^a+\mathcal {N}^a, \end{aligned}$$

defined by

51a $$\begin{aligned} {\mathcal G}^a&:=-\int \hbox {d}^3x \partial _\nu F_{\nu 0}^a=\int _{S_\infty } d \sigma _i F_{0i}, \end{aligned}$$

51b $$\begin{aligned} \mathcal {N}^a&:=i \left\{ Q_\mathrm{BRST}, \int \hbox {d}^3x\, (D_0\bar{c})^a \right\} \nonumber \\&= \left\{ \bar{Q}_\mathrm{BRST}, \int \hbox {d}^3x\, \pi _{\bar{c}} \right\} , \end{aligned}$$

are individually conserved. Here $\pi _{\bar{c}}$ is canonically conjugate to the antighost $\bar{c}$. Along the lines of the argument in Abelian gauge theories following Eq. (31), Kugo and Ojima showed [1, 2] that all physical operators are colorless and commute with $Q^a$ if two conditions are fulfilled:

(i) The Lorentz-invariant function $f_\mathrm{LCG}$ defined by the transverse correlation function,
  52 $$\begin{aligned} \left\langle A^a_\sigma (y)\, F^b_{\nu \mu }(x) \right\rangle _\mathcal {FT}\!=\! - i \delta ^{ab}(\delta _{\sigma \mu } p_\nu \!-\! \delta _{\sigma \nu }p_\mu ) \frac{f_\mathrm{LCG}(p^2)}{p^2},\nonumber \\ \end{aligned}$$
  must vanish at $p^2=0$, implying the absence of a massless vector boson in the adjoint representation of the group.
(ii) The function $u_\mathrm{LCG}$, defined by
  53 $$\begin{aligned} -i\left\langle A_{\sigma }^a(y)\,s\bar{s} A_\mu ^b(x) \right\rangle&= i\left\langle (D_\sigma c)^a(y)\,(D_\mu \bar{c} )^b(x) \right\rangle _\mathcal {FT}\nonumber \\&= \delta ^{ab}\left( T_{\sigma \mu }\,u_\mathrm{LCG}(p^2) - L_{\sigma \mu }\right) ,\nonumber \\ \end{aligned}$$
  must assume the value, $u(p^2\rightarrow 0)=-1$, in the infrared limit.

As in the Abelian case, condition (52) with $f_\mathrm{LCG}(0) = 0$, also holds in a non-Abelian Higgs phase, in which the vector bosons is massive. The infrared behavior of $u_\mathrm{LCG}(p^2)$ thus distinguishes between the Higgs and confinement phase in LCG. In terms QEoM of the gauge boson propagator this distinction may be reformulated as: if the Kugo–Ojima criterion is fulfilled, that is, if

54 $$\begin{aligned} u_\mathrm{LCG}(0)=-1 \quad \hbox {and}\quad f_\mathrm{LCG}(0)=0, \end{aligned}$$

the QEoM of the vector boson propagator,

55 $$\begin{aligned} \delta ^{ab}\delta _{\mu \sigma }\delta (x-y)&= \left\langle A_{\sigma }^a(y)\,\frac{\delta S_\mathrm{LCG}}{\delta A_{\mu }^b(x)} \right\rangle \end{aligned}$$

56 $$\begin{aligned}&= \left\langle A_{\sigma }^a(y)\,(-\partial _\nu F_{\nu \mu }^b - j_\mu ^{\mathrm{LCG}\,b}(x) \right\rangle \nonumber \\&\quad + i\left\langle A_{\sigma }^a(y)\,s\bar{s} A_\mu ^b(x) \right\rangle , \end{aligned}$$

at vanishing momentum is saturated by *unphysical* states only. By contrast, for $f_\mathrm{LCG}(0)=0$ and $u_\mathrm{LCG}(0)\ne -1$ the theory may describe a Higgs phase in which *physical* states contribute to the saturation of the transverse part of the current matrix element $\left\langle A_{\sigma }^a(y)\, j_\mu ^{\mathrm{LCG}\,b})(x) \right\rangle$ at vanishing momentum. Since one cannot be sure that physical states contribute when $u_\mathrm{LCG}(0)\ne -1$, the criterion of Kugo and Ojima of Eq. (54) is a *sufficient* criterion for confinement [2].

The longitudinal part of the correlation function in Eq. (53) is uniquely determined by the equation of motion of the ghost field. It saturates the longitudinal part of Eq. (56) for all momenta and implies that the current matrix element in LCG is transverse.

If the Kugo–Ojima criterion is fulfilled, the matrix element of the BRST-exact part of the (generalized) color current saturates the transverse gluonic QEoM of LCG at low momentum. Assuming the BRST symmetry is unbroken, Kugo and Ojima proved that the physical sector in this case is colorless [1, 2, 76]. In a Higgs phase, the physical, albeit massive, vector boson would contribute to the transverse part of the gluonic QEoM at vanishing momentum and it would not be saturated by unphysical states only.

Since color confinement is not compatible with physical states in the adjoint color representation, an alternative criterion for the confining phase, but one that includes the essential information, is the saturation of the gluonic QEoM by unphysical degrees of freedom in the infrared. We now investigate whether this proposal can be applied to a wider class of gauges.

### Saturation and confinement in generalized linear covariant gauges (GLCG) 

Let us therefore next examine saturation of the gluonic QEoM in Generalized Linear Covariant gauges (GLCG) given by the Lagrangian [79, 80]

57 $$\begin{aligned} \mathcal L_\mathrm{GLCG}&= \mathcal L_\mathrm{YM}+s_\alpha \left( \bar{c}^a \left( \frac{\xi }{2} b^a- i\partial _\mu A^a_\mu \right) \right) \nonumber \\&= \mathcal L_\mathrm{YM}+ \frac{\xi }{2} b^2 - i \partial _\mu A_\mu ^a b^a - i \alpha \left( D_\mu \bar{c}\right) ^a \partial _\mu c^a \nonumber \\&\quad - i \bar{\alpha }\partial _\mu \bar{c}^a \left( D_\mu c\right) ^a + \frac{\alpha \bar{\alpha } \xi }{2} \left( \bar{c}\! \times \! c \right) ^{2} , \end{aligned}$$

with $\alpha + \bar{\alpha } \!=\! 1$. The Lagrangian of Eq. (57) interpolates between LCG ($\alpha = 0$), its Faddeev–Popov conjugate ($\alpha = 1$), and the ghost–antighost symmetric gauge at $\alpha =$ $\bar{\alpha }= \frac{1}{2}$. The generalized Kugo–Ojima confinement scenario for this Lagrangian is discussed in [81]. For any value of the gauge parameters $\alpha$ and $\xi$, $\mathcal L_\mathrm{GLCG}$ is globally color symmetric and invariant under the nilpotent BRST and anti-BRST transformations,

58 $$\begin{aligned}&s_\alpha A_\mu ^a = D_\mu c^a, \nonumber \\&s_\alpha c^a = -\frac{1}{2} \left( c\! \times \! c \right) ^{a} ,\end{aligned}$$

59 $$\begin{aligned}&s_\alpha \bar{c} = b^a - \alpha \left( \bar{c}\! \times \! c \right) ^{a} , \nonumber \\&s_\alpha b^a = -\alpha \left( c\! \times \! b \right) ^{a} + \frac{\alpha \bar{\alpha }}{2} \left( \bar{c}\! \times \! \left( c\! \times \! c \right) ^{} \right) ^{a} ;\nonumber \\&\bar{s}_\alpha A_\mu ^a = D_\mu \bar{c} ^a, \nonumber \\&\bar{s}_\alpha \bar{c}^a = -\frac{1}{2} \left( \bar{c}\! \times \! \bar{c} \right) ^{a} , \nonumber \\&\bar{s}_\alpha c = - b^a - \bar{\alpha }\left( \bar{c}\! \times \! c \right) ^{a} , \\&\bar{s}_\alpha b^a = - \bar{\alpha } \left( \bar{c}\! \times \! b \right) ^{a} + \frac{\alpha \bar{\alpha }}{2}\left( \left( \bar{c}\! \times \! \bar{c} \right) ^{} \! \times \! c \right) ^{a} .\nonumber \end{aligned}$$

The ghost–antighost symmetric gauge $\alpha =\bar{\alpha }=\frac{1}{2}$ possesses an additional continuous global $\hbox {SL}(2,R)$ symmetry generated by $\Pi _{\mp }$ and the ghost number $\Pi$,

60 $$\begin{aligned} \Pi _+&= \int \hbox {d}^4x \; c(x)\frac{\delta }{\delta \bar{c}(x)},\nonumber \\ \Pi _-&= \int \hbox {d}^4x\; \bar{c}(x)\frac{\delta }{\delta c(x)},\ \ \ [\Pi _+,\Pi _-]=2 \Pi . \end{aligned}$$

One verifies that the BRST and anti-BRST charges $Q_\mathrm{BRST}$ and $\bar{Q}_\mathrm{BRST}$, which generate the transformations of Eqs. (58) and (59), anticommute [79], and that the graded algebra of $\{Q_\mathrm{BRST},\bar{Q}_\mathrm{BRST}, \Pi _+,\Pi _-,\Pi \}$ closes in ghost–antighost symmetric gauges. The space of physical operators $\mathfrak {P}$ is defined as in Eq. (17).

Due to the invariance of Eq. (57) under global color transformations, the Noether currents,

61 $$\begin{aligned} j_\mu ^{\mathrm{GLCG}\,a}&= \left( A_\nu \! \times \! (F_{\nu \mu } \!+\! i b \delta _{\mu \nu }) \right) ^{a} \!\!-\! i \left( c\! \times \! (\alpha D_\mu \bar{c} + \bar{\alpha } \partial _\mu \bar{c}) \right) ^{a} \nonumber \\&+ i \left( \bar{c}\! \times \! (\bar{\alpha } D_\mu c + \alpha \partial _\mu c ) \right) ^{a} , \end{aligned}$$

are conserved. These currents depend explicitly on the gauge parameter $\alpha$. They are part of the gluonic QEoM, which here takes the form,

62 $$\begin{aligned} \delta ^{ab}_{\mu \sigma }\delta (x-y)&=\left\langle A_{\sigma }^a(y)\,\frac{\delta S_\mathrm{GLCG}}{\delta A_{\mu }^b(x)} \right\rangle \nonumber \\&=- \left\langle A_{\sigma }^a(y)\,(\partial _\nu F_{\nu \mu } + j_{\mu }^\mathrm{GLCG})^b(x) \right\rangle \nonumber \\&\quad + \left\langle A_{\sigma }^a(y)\,(is_\alpha \bar{s}_\alpha A_\mu )^b(x) \right\rangle . \end{aligned}$$

The last term in Eq. (62) again involves only unphysical excitations and is of the same form as in the LCG studied above,

63 $$\begin{aligned}&\left\langle A_{\sigma }^a(y)\,(is_\alpha \bar{s}_\alpha A_\mu )^b(x) \right\rangle _\mathcal {FT}\nonumber \\&\quad = -i\left\langle (D_{\sigma }c)^a(y)\, (D_\mu \bar{c})^b(x) \right\rangle _\mathcal {FT}\nonumber \\&\quad = \delta ^{ab}\left( L_{\sigma \mu }-T_{\sigma \mu }\,u_\mathrm{GLCG}(p^2) \right) . \end{aligned}$$

The equation of motion of the ghost by itself does not suffice to determine the longitudinal part of Eq. (63). Instead one has

64 $$\begin{aligned} i(\partial _\mu D_\mu \bar{c})^a=\frac{\delta S_\mathrm{GLCG}}{\delta c^a}- \frac{\xi \bar{\alpha }}{2} s (\bar{c}\times \bar{c})^a. \end{aligned}$$

Since $\langle A_\mu ^a \rangle = 0$ and the BRST transformation is nilpotent, Eq. (64) determines the longitudinal part of the correlation function in Eq. (63). As in LCG, unphysical degrees of freedom saturate the longitudinal part of the gluonic QEoM in Eq. (62), and the current matrix element is transverse.

The form factor, $f_\mathrm{GLCG}(p^2)$, is defined as in Eq. (52), and the same discussion as in Sect. 3.1 applies. The theory describes a Coulomb phase with a massless vector boson only if $f_\mathrm{GLCG}(0)\ne 0$. The transverse part of the correlator in Eq. (63) defines a form factor $u_\mathrm{GLCG}(p^2)$ whose value in the infrared can be used as a criterion in these gauges. The transverse gluonic QEoM is saturated by unphysical degrees of freedom in the infrared and the phase is confining if $u_\mathrm{GLCG}(0)=-1$. One thus formally has the same confinement criterion as in LCG [81]. However, although the unphysical correlation functions in Eqs. (63) and (53) formally look similar, unphysical correlations differ, and in general $u_\mathrm{GLCG}(p^2)\ne u_\mathrm{LCG}(p^2)$ if $\alpha \ne 0$. The confinement criterion stated above asserts that these functions in the confining phase may coincide at $p^2=0$ in any gauge parametrized by $(\alpha ,\xi )$. We next turn to covariant gauges that explicitly break global color invariance to the Cartan subgroup.

### Saturation and confinement in maximal Abelian gauges (MAG)

An equivariant BRST construction allows one to partially localize a non-Abelian gauge theory to an equivalent Abelian model with the same gauge-invariant correlators. This partial localization is possible on the lattice [41, 82, 83] as well as in the continuum [44, 84, 85] and defines the continuum theory as the critical limit of a lattice model with the same global symmetries. The equivariant construction may be viewed as a partial gauge fixing that leaves the Abelian Cartan subgroup free, hence Maximal Abelian Gauge. The resulting Abelian gauge theory has the same gauge-invariant physical correlation functions as the non-Abelian model and the residual Abelian gauge symmetry of MAG can be dealt with as in any Abelian gauge theory. An $\hbox {SU}(N)$ gauge theory in MAG presents itself as an Abelian $({U(1)})^{N-1}$ gauge theory that is asymptotically free but retains typical Abelian Ward identities. In the following we investigate how a confining phase may manifest itself in this Abelian gauge theory.

In MAG one discriminates between the Cartan subgroup and the coset space. For an $\hbox {SU}(N)$ gauge theory the mutually commuting (Hermitian) generators of the Cartan subgroup will be denoted by $\{T^i; [T^i,T^j]=0\ \forall i,j=1,\dots ,N-1\}$, whereas the remaining $N(N-1)$ coset generators carry Latin indices from the beginning of the alphabet $\{T^a; a=1,\dots ,N(N-1)\}$. The non-Abelian $\hbox {SU}(N)$ connection is decomposed as $\mathcal A_\mu =A_\mu ^i T^i+ B_\mu ^a T^a$ and the field strength tensor may similarly be decomposed into Cartan and coset components, $\mathcal F_{\mu \nu } = f_{\mu \nu }^i T^i + F_{\mu \nu }^{a}T^a$ with

65a $$\begin{aligned} f_{\mu \nu }^i&= \partial _\mu A_\nu ^i - \partial _\nu A_\mu ^i + \left( B_\mu \! \times \! B_\nu \right) ^{i} \quad \text { and } \end{aligned}$$

65b $$\begin{aligned} F_{\mu \nu }^a&= (\mathsf {D}_\mu B_\nu )^a - (\mathsf {D}_\nu B_\mu )^a + \left( B_\mu \! \times \! B_\nu \right) ^{a} . \end{aligned}$$

Here and in the following the covariant derivative with respect to the Cartan gluons in the adjoint representation is denoted by $\mathsf {D}_\mu ^{ab}$; see Appendix A. The $\hbox {SU}(N)$ Yang–Mills Lagrangian in the components of Eq. (65) reads

66 $$\begin{aligned} \mathcal L_\mathrm{YM}= \frac{1}{4} f_{\mu \nu }^i f_{\mu \nu }^i + \frac{1}{4} F_{\mu \nu }^a F_{\mu \nu }^a. \end{aligned}$$

Although a more general discussion is possible, we for simplicity consider the gauge group $\hbox {SU}(2)$ in the following. It illustrates our main points and connects to our considerations in Sect. 2. The Cartan subalgebra in this case is one dimensional and we suppress its index. The coset space is two dimensional with components $a=1,2$.

The distinction between Abelian and coset degrees of freedom is accomplished by the “gauge-fixing” part of the MAG Lagrangian,

67 $$\begin{aligned} \mathcal L_\mathrm{GF}^\mathrm{MAG}&= \frac{i}{2} s_\varepsilon \bar{s}_\varepsilon \left( B_\mu ^a B_\mu ^a - i \lambda \bar{c} ^a c^a \right) \nonumber \\&= \frac{\lambda }{2} \eta ^a \eta ^a - i \eta ^a(\mathsf {D}_\mu B_\mu )^a - i (\mathsf {D}_\mu \bar{c})^a(\mathsf {D}_\mu c)^a \nonumber \\&\quad + i \left( B_\mu \! \times \! \bar{c} \right) ^{} \left( B_\mu \! \times \! c \right) ^{} + \frac{\lambda }{2}\,\left( \bar{c}\! \times \! c \right) ^{2} . \end{aligned}$$

$\mathcal L_\mathrm{GF}^\mathrm{MAG}$ is obtained using the equivariant BRST and anti-BRST transformations [41, 44],

68a $$\begin{aligned} s_\varepsilon A_\mu&= \left( B_\mu \! \times \! c \right) ^{} , \quad \bar{s}_\varepsilon A_\mu =\left( B_\mu \! \times \! \bar{c} \right) ^{} , \end{aligned}$$

68b $$\begin{aligned} s_\varepsilon B^a_\mu&= (\mathsf {D}_\mu c)^a, \quad \bar{s}_\varepsilon B^a_\mu = (\mathsf {D}_\mu \bar{c})^a, \end{aligned}$$

68c $$\begin{aligned} s_\varepsilon c^a&= 0, \quad \bar{s}_\varepsilon \bar{c}^a = 0, \end{aligned}$$

68d $$\begin{aligned} s_\varepsilon \bar{c}^a&= \eta ^a, \quad \bar{s}_\varepsilon c^a = -\eta ^a, \end{aligned}$$

68e $$\begin{aligned} s_\varepsilon \eta ^a&= \frac{1}{2} \left( \bar{c}\! \times \! \left( c\! \times \! c \right) ^{} \right) ^{a} , \quad \bar{s}_\varepsilon \eta ^a = \frac{1}{2} \left( \left( \bar{c}\! \times \! \bar{c} \right) ^{} \! \times \! c \right) ^{a} , \end{aligned}$$

which generate infinitesimal gauge transformations in the coset space $\hbox {SU}(2)/U(1)$ with parameters $c^a(x)$ and $\bar{c}^a(x)$. $\mathcal L_\mathrm{YM}$ and covariantly coupled matter are invariant under these transformations. Contrary to LCG, $\hbox {SU}(2)$ in MAG has two (anti-)ghosts only. The Lagrangian

69 $$\begin{aligned} \mathcal L^{U(1)}_\mathrm{MAG} = \mathcal L_\mathrm{YM}+ \mathcal L_\mathrm{GF}^\mathrm{MAG} \end{aligned}$$

defines an ${U(1)}$ invariant gauge theory. It includes exotic ${U(1)}$-charged $B^a_\mu , c^a$ and $\bar{c}^a$ “matter” and is invariant under the equivariant BRST and anti-BRST transformations of Eq. (68) and under infinitesimal local ${U(1)}$ transformations,

70 $$\begin{aligned}&A_\mu \rightarrow \partial _\mu \theta ,\quad B_\mu ^a\rightarrow \left( B_\mu \! \times \! \theta \right) ^{a} ,\quad c^a \rightarrow \left( c\! \times \! \theta \right) ^{a} ,\nonumber \\&\quad \bar{c}^a \rightarrow \left( \bar{c}\! \times \! \theta \right) ^{a} \,\text {and}\quad \eta ^a\rightarrow \left( \eta \! \times \! \theta \right) ^{a} . \end{aligned}$$

Analogous to Eq. (15) one can define the equivariant BRST and anti-BRST charges, $Q_{\varepsilon BRST}$ and $\bar{Q}_{\varepsilon BRST}$. The Lagrangian (67) and the algebra of $Q_{\varepsilon BRST}$ and $\bar{Q}_{\varepsilon BRST}$ are ghost–antighost symmetric. In addition to the ghost number $\Pi$, one thus has the complete $\hbox {SL}(2,R)$ algebra of charges typical of ghost–antighost symmetric gauges. However, in MAG the $\Pi _\mp$ of Eq. (60) involve only two coset (anti-)ghosts rather than the three of an $\hbox {SU}(2)$ gauge theory in ghost–antighost symmetric GLCG.

The equivariant BRST transformations (68) are *not* nilpotent. $s_\varepsilon ^2$, $\bar{s}_\varepsilon ^2$ and $\frac{1}{2}\left\{ s_\varepsilon , \bar{s}_\varepsilon \right\}$ generate ${U(1)}$ transformations with a bosonic parameter $\theta (x) = \tfrac{1}{2} \left( c\! \times \! c \right) ^{} , \tfrac{1}{2} \left( \bar{c}\! \times \! \bar{c} \right) ^{} \,\text {and}\,\frac{1}{2}\left( \bar{c}\! \times \! c \right) ^{}$, respectively. However, $s_\varepsilon ^2\mathcal {O}= \bar{s}_\varepsilon ^2\mathcal {O}= \left\{ s_\varepsilon , \bar{s}_\varepsilon \right\} \mathcal {O}= 0$ for any operator $\mathcal {O}$ invariant under the ${U(1)}$ transformations of Eq. (70). The equivariant BRST algebra thus reduces to the usual nilpotent BRST algebra on the set of ${U(1)}$ invariant functionals only.

The residual ${U(1)}$ symmetry of the Lagrangian defined by Eq. (69) can be fixed to any gauge. To allow for comparison with Sect. 2 we here choose a linear covariant gauge

71 $$\begin{aligned} \mathcal L_\mathrm{GF}^{U(1)}= \frac{\xi }{2}b^2-ib\partial _\mu A_\mu \end{aligned}$$

with gauge-fixing parameter $\xi$ and (uncharged) auxiliary field $b$. $b$ is taken to be invariant under $s_\varepsilon$ and $\bar{s}_\varepsilon$, $s_\varepsilon b = \bar{s}_\varepsilon b = 0$.

We thus consider the $\hbox {SU}(2)$-Yang–Mills theory in MAG specified by the action $\mathcal {S}_\mathrm{MAG}= \int \!\hbox {d}^4x \mathcal L_\mathrm{MAG}(x)$ with,

72 $$\begin{aligned} \mathcal L_\mathrm{MAG} = \mathcal L_\mathrm{YM}+ \mathcal L_\mathrm{GF}^\mathrm{MAG} + \mathcal L_\mathrm{GF}^{U(1)}. \end{aligned}$$

The ${U(1)}$ gauge fixing $\mathcal L_\mathrm{GF}^{U(1)}$ of Eq. (72) not only explicitly breaks the local ${U(1)}$-gauge symmetry but global symmetries as well. $\mathcal L_\mathrm{GF}^{U(1)}$ is symmetric under global ${U(1)}$-transformations, but it breaks the global equivariant BRST, anti-BRST and $\hbox {SL}(2)$ symmetries explicitly. For any, not necessarily local, operator $\mathcal {O}$ one has the Ward identities,

73a $$\begin{aligned} \left\langle \delta _x \mathcal {O} \right\rangle&= \left\langle \mathcal {O}\ \delta _x \mathcal {S}_\mathrm{MAG} \right\rangle =i\left\langle \mathcal {O}\ \partial ^2 b(x) \right\rangle \end{aligned}$$

73b $$\begin{aligned} \left\langle s_\varepsilon \mathcal {O} \right\rangle&= \left\langle \mathcal {O}\ s_\varepsilon \mathcal {S}_\mathrm{MAG} \right\rangle =i\left\langle \mathcal {O}\ \int (\partial _\mu b)s_\varepsilon A_\mu \right\rangle \end{aligned}$$

73c $$\begin{aligned} \left\langle \bar{s}_\varepsilon \mathcal {O} \right\rangle&= \left\langle \mathcal {O}\ \bar{s}_\varepsilon \mathcal {S}_\mathrm{MAG} \right\rangle =i\left\langle \mathcal {O}\ \int (\partial _\mu b)\bar{s}_\varepsilon A_\mu \right\rangle , \end{aligned}$$

where

74 $$\begin{aligned} \delta _x \mathcal {O}&= \left( \partial _\mu \frac{\delta }{\delta A_\mu (x)} + B_\mu (x)\times \frac{\delta }{\delta B_\mu (x)} + c(x)\times \frac{\delta }{\delta c(x)}\right. \nonumber \\&\left. + \bar{c}(x)\times \frac{\delta }{\delta \bar{c}(x)} + \eta (x)\times \frac{\delta }{\delta \eta (x)}\right) \mathcal {O}, \end{aligned}$$

generates a local ${U(1)}$ gauge transformation at $x$. Defining the set $\mathfrak {W}$ of ${U(1)}$-invariant operators,

75 $$\begin{aligned} \mathcal {O}\in \mathfrak {W}\Leftrightarrow \delta _x \mathcal {O}=0, \end{aligned}$$

we show in Appendix B that

76 $$\begin{aligned} \left\langle s_\varepsilon \mathcal {O} \right\rangle =\left\langle \bar{s}_\varepsilon \mathcal {O} \right\rangle =0\ \ \quad \text {for all } \mathcal {O}\in \mathfrak {W}. \end{aligned}$$

On the set $\mathfrak {W}$ of ${U(1)}$ invariant operators of the equivalent *Abelian* gauge theory one thus recovers $s_\varepsilon$ and $\bar{s}_\varepsilon$ as nilpotent BRST symmetries. One then can define the set of physical operators $\mathfrak {P}\subseteq \mathfrak {W}$ of the underlying non-Abelian $\hbox {SU}(2)$ gauge theory by the equivariant cohomology,

77 $$\begin{aligned} \mathfrak {P}&= \{ \mathcal {O}\in \mathfrak {W}; \left[ Q_{\varepsilon \mathrm{BRST}} , \mathcal {O} \right] =0\ \text {and }[\mathcal {O},\Pi ]=0 \} \nonumber \\&/ \{ \left[ Q_{\varepsilon \mathrm{BRST}} , \mathcal {O} \right] ;\mathcal {O}\in \mathfrak {W}\ \text {and}\ [\mathcal {O},\Pi ]=\mathcal {O}\}. \end{aligned}$$

The conserved ${U(1)}$ current of the Cartan subalgebra of $\hbox {SU}(2)$ in MAG is

78a $$\begin{aligned} j^\mathrm{MAG}_\mu&= \left( B_\nu \! \times \! F_{\nu \mu } \right) ^{} + i \left( B_\mu \! \times \! \eta \right) ^{} +i \left( \bar{c}\! \times \! D_\mu c \right) ^{} \nonumber \\&\quad - i \left( D_\mu \bar{c}\! \times \! c \right) ^{} , \end{aligned}$$

and can be rewritten in the form,

78b $$\begin{aligned} j^\mathrm{MAG}_\mu&= i \left[ s_\varepsilon , \bar{s}_\varepsilon \right] A_\mu +B_\nu \times ( F_{\nu \mu } - i \delta _{\mu \nu } \eta )\in \mathfrak {W}. \end{aligned}$$

In fact, each term in Eq. (78b) separately is an element of $\mathfrak {W}$, but $i \left[ s_\varepsilon , \bar{s}_\varepsilon \right] A_\mu$ does not create *physical* states.

The QEoM of the Cartan gluon propagator depends on the conserved Abelian Noether current of Eq. (78b) as in the Abelian gauge theory studied in Sect. 2,

79 $$\begin{aligned} \delta _{\sigma \mu }\delta (x-y)&= \left\langle A_{\sigma }(y)\,\frac{\delta \mathcal {S}_\mathrm{MAG}}{\delta A_{\mu }(x)} \right\rangle \nonumber \\&= -\left\langle A_\sigma (y)\,\left( \partial _\nu f_{\nu \mu } + j^\mathrm{MAG}_\mu \right) (x) \right\rangle \nonumber \\&\quad + \left\langle A_\sigma (y)\,i\partial _\mu b(x) \right\rangle . \end{aligned}$$

As for an unbroken Abelian gauge theory, the last term of Eq. (79) saturates the longitudinal part of the gluonic QEoM because of the Abelian Ward identity of Eq. (73a),

80 $$\begin{aligned} \partial _\sigma \delta (x-y)&= \left\langle \delta _x A_\sigma (y) \right\rangle =i\left\langle A_\sigma (y)\, \partial ^2 b(x) \right\rangle \nonumber \\&\Rightarrow \left\langle A_\sigma (y)\, i\partial _\mu b(x) \right\rangle _\mathcal {FT}=L_{\sigma \mu }. \end{aligned}$$

The first correlator in Eq. (79) is transverse due to the antisymmetry of $f_{\mu \nu }$ and the current matrix element thus is transverse as well,

81 $$\begin{aligned} \left\langle A_\sigma (y)\,j^\mathrm{MAG}_\mu (x) \right\rangle _\mathcal {FT}=(f_\mathrm{MAG}(p^2)-1)T_{\mu \nu }. \end{aligned}$$

The functions $f_\mathrm{MAG},\, u_\mathrm{MAG},\, h_\mathrm{MAG},$ and $\ell _\mathrm{MAG}$ are defined from the correlators

82a $$\begin{aligned}&-\left\langle A_\sigma (y)\, \partial _\nu f_{\nu \mu } (x) \right\rangle _\mathcal {FT}= f_\mathrm{MAG}(p^2) T_{\sigma \mu }\end{aligned}$$

82b $$\begin{aligned}&i\left\langle A_\sigma (y)\, \left[ s_\varepsilon , \bar{s}_\varepsilon \right] A_\mu (x) \right\rangle _\mathcal {FT}= u_\mathrm{MAG}(p^2) T_{\sigma \mu } \nonumber \\&\quad + \ell _\mathrm{MAG}(p^2) L_{\sigma \mu } \end{aligned}$$

82c $$\begin{aligned}&-\left\langle A_\sigma (y)\, \left( B_\nu \! \times \! (F_{\nu \mu } - \delta _{\mu \nu } i \eta ) \right) ^{} \right\rangle _\mathcal {FT}= h_\mathrm{MAG}(p^2) T_{\sigma \mu }\nonumber \\&\quad +\ell _\mathrm{MAG}(p^2) L_{\sigma \mu }, \end{aligned}$$

and the transverse part of Eq. (79) yields the constraint

83 $$\begin{aligned} f_\mathrm{MAG}(p^2) + h_\mathrm{MAG}(p^2) = 1+ u_\mathrm{MAG}(p^2). \end{aligned}$$

As in LCG and GLCG, the transverse gluonic QEoM is saturated by unphysical degrees of freedom and the Cartan color charge of physical states vanishes when the Kugo–Ojima-like criterion

84 $$\begin{aligned} f_\mathrm{MAG}(0)=0 \quad \hbox { and }\quad u_\mathrm{MAG}(0)=-1, \end{aligned}$$

holds since it implies that $h_\mathrm{MAG}(0)=0$. The conditions (84) guarantee saturation of the gluonic QEoM in the infrared by unphysical degrees of freedom in MAG and implies that physical states are colorless. Equation (81) together with Eq. (84) imply that this scenario can only be realized in MAG if unphysical degrees of freedom created by the *conserved Abelian current* $j_\mu ^\mathrm{MAG}$ saturate the QEoM of the Abelian propagator at low momenta. From the point of view of the Abelian gauge theory, saturation of the transverse equation at low momenta in confinement and Higgs phases thus is similar. In MAG the only difference is that whereas some *physical* degrees of freedom contribute to the matrix element of the current at vanishing momentum in the Higgs phase, only *unphysical* states contribute in the confinement phase. The current saturates the QEoM of the Abelian propagator at low energies in the Higgs phase described by Eq. (37) as well as in the confinement phase of the $\hbox {SU}(2)$ gauge theory in MAG defined by Eq. (69). This supports the idea that the phases and the two Abelian models describing them are dual [8].

In this context it is interesting to consider the condition $f_\mathrm{MAG}(0) = 0$ more closely. The analogous condition implies a massive *physical* vector boson in the Abelian theory described by Eq. (34). The Cartan gluon on the other hand is not a physical asymptotic state in the confinement phase and it has been conjectured [49] that the Abelian propagator in this case may even be enhanced in the infrared. That this scenario can be reconciled with the criterion of Eq. (84) rests on the definition Eq. (65a) of the Abelian field strength tensor $f_{\mu \nu }$. Equation (82a) implies

85 $$\begin{aligned}&f_\mathrm{MAG}(p^2) T_{\sigma \mu } = p^2 T_{\mu \nu } \left\langle A_\sigma (y) \,A_\nu (x) \right\rangle _\mathcal {FT}\nonumber \\&\quad - \left\langle A_\sigma (y) \ \partial _\nu \left( B_\nu \! \times \! B_\mu \right) ^{} \!(x) \right\rangle _\mathcal {FT}. \end{aligned}$$

Although a massive Abelian vector boson allows one to fulfill $f_\mathrm{MAG}(0)=0$, the last correlator of Eq. (85) prohibits one from asserting that the diagonal gluon propagator *has to be* suppressed at low momenta.

Introducing the function $\alpha _\mathrm{MAG}(p^2)$,

86 $$\begin{aligned} \left\langle A_\sigma (y) \ \left( B_\nu \! \times \! B_\mu \right) ^{} \!(x) \right\rangle _\mathcal {FT}&= -i (\delta _{\sigma \nu } p_\mu -\delta _{\sigma \mu }p_\nu ) \nonumber \\&\alpha _\mathrm{MAG}(p^2),\nonumber \\ \end{aligned}$$

Equation (85) states that

87 $$\begin{aligned} f_\mathrm{MAG}(p^2) = p^2 \left( {\textstyle \frac{1}{3}}T_{\mu \nu } \left\langle A_\mu (y) \,A_\nu (x) \right\rangle _\mathcal {FT}- \alpha _\mathrm{MAG}(p^2)\right) . \nonumber \\ \end{aligned}$$

If the Cartan gluon correlator is infrared enhanced, Eq. (87) determines only the infrared singular behavior of $\alpha _\mathrm{MAG}(p^2)$ when $f_\mathrm{MAG}(0)=0$.

To gain some more information about these functions, we define a ${U(1)}$-invariant transverse field strength,

88 $$\begin{aligned} G_{\mu \nu }=\partial _\mu A_\nu -\partial _\nu A_\mu . \end{aligned}$$

It is not an invariant of the equivariant BRST (or anti-BRST) and, in contrast to the ${U(1)}$ gauge theory considered in Sect. 2, is not a physical operator of the $\hbox {SU}(2)$ gauge theory. The function $u_\mathrm{MAG}(p^2)$ defined in Eq. (82b) also describes the correlation functions,

89 $$\begin{aligned}&u_\mathrm{MAG}(p^2)(\delta _{\rho \mu }p_\sigma -\delta _{\sigma \mu }p_\rho ) = \left\langle G_{\rho \sigma }(y)\, \left[ s_\varepsilon , \bar{s}_\varepsilon \right] A_\mu (x) \right\rangle _\mathcal {FT}\nonumber \\&\quad =\left\langle \bar{s}_\varepsilon G_{\rho \sigma }(y)\, s_\varepsilon A_\mu (x) \right\rangle _\mathcal {FT}- \left\langle s_\varepsilon G_{\rho \sigma }(y)\, \bar{s}_\varepsilon A_\mu (x) \right\rangle _\mathcal {FT},\nonumber \\ \end{aligned}$$

where Eq. (76) was used since $\{G_{\mu \nu },s_\varepsilon A_\mu ,\bar{s}_\varepsilon A_\mu \}\subset \mathfrak {W}$. Using the definition in Eq. (88) and exploiting Poincaré invariance, Eq. (89) implies,

90 $$\begin{aligned}&u_\mathrm{MAG}(p^2) T_{\mu \nu }+v_\mathrm{MAG}(p^2) L_{\mu \nu }=2i\left\langle \bar{s}_\varepsilon A_\nu (y)\,s_\varepsilon A_\mu (x) \right\rangle _\mathcal {FT}\nonumber \\&\quad =2i\left\langle \left( B_\nu \! \times \! \bar{c} \right) ^{} (y)\, \left( B_\mu \! \times \! c \right) ^{} (x) \right\rangle _\mathcal {FT}, \end{aligned}$$

in close analogy to Eq. (53) in general covariant gauges. Neither Eq. (89) nor the equations of motion constrain the longitudinal function $v_\mathrm{MAG}(p^2)$ in this case. Since Eq. (76) holds only for ${U(1)}$-invariant functionals in $\mathfrak {W}$, $v_\mathrm{MAG}(p^2)$ also need not be related to $\ell _\mathrm{MAG}(p^2)$ defined by Eq. (82b).^1^

We next study signatures of the confining phase in a gauge that is not covariant.

### Saturation and confinement in non-Abelian coulomb gauge (CG)

Coulomb gauge breaks manifest Lorentz covariance by treating timelike and spacelike gluons differently. We here study to what extent the Kugo–Ojima criterion depends on a manifestly Lorentz-invariant gauge condition. Coulomb gauge is described by the Lagrangian,

91 $$\begin{aligned} \mathcal L_C&= \mathcal L_\mathrm{YM}-i s\left( \bar{c}^a \partial _i A_i^a \right) \nonumber \\&= \mathcal L_\mathrm{YM}- i b^a \partial _i A_i^a - i \partial _i\bar{c}^a (D_ic)^a, \end{aligned}$$

where Latin indices denote spatial components of a Lorentz-vector, $i,j,\ldots = 1,2,3$. The BRST transformations are the same as in Eq. (46),

92 $$\begin{aligned}&s A_0^a = (D_0 c)^a, \quad s A_i^a = (D_i c)^a, \quad sc^a = -\frac{1}{2} \left( c\! \times \! c \right) ^{a} ,\nonumber \\&s \bar{c} ^a = b^a, \quad sb^a = 0. \end{aligned}$$

The BRST charge in Coulomb gauge can be written in terms of Gauss’s law [86]

93 $$\begin{aligned} Q_\mathrm{BRST} = - \int \! \hbox {d}^3x\, c^a (D_i F_{i0})^a = \int \! \hbox {d}^3x \,c^a \frac{\delta S_C}{\delta A_0^a}. \end{aligned}$$

The anti-BRST transformations and the corresponding charge may be defined analogously and the set of physical operators again is given by the BRST cohomology of Eq. (17). Coulomb gauge manifestly preserves global color symmetry and the color currents

94a $$\begin{aligned} {j^C_0}^a&= \left( A_i\! \times \! F_{i0} \right) ^{a} ,\end{aligned}$$

94b $$\begin{aligned} {j^C_i}^a&= \left( A_0\! \times \! F_{0i} \right) ^{a} + \left( A_j\! \times \! F_{ji} \right) ^{a} + \left( A_i\! \times \! b \right) ^{a} \nonumber \\&- i \left( c\! \times \! \partial _i \bar{c} \right) ^{a} + i \left( \bar{c}\! \times \! D_i c \right) ^{a} , \end{aligned}$$

are conserved. The absence of manifest Lorentz invariance in Coulomb gauge implies two distinct gluonic QEoMs. The QEoM of the time component reads

95 $$\begin{aligned} \delta ^{ab}&= \left\langle A_0^b(y)\,\frac{\delta S_C}{\delta A_0^a(x)} \right\rangle _\mathcal {FT}\nonumber \\&= \left\langle A_0^b(y)\, \left( -\partial _iF_{i0}^a(x) - j^{C\,a}_0(x)\right) \right\rangle _\mathcal {FT}\nonumber \\&= -\left\langle A_0^b(y)\,D_iF_{i0}^a(x) \right\rangle _\mathcal {FT}. \end{aligned}$$

Since all physical states satisfy Gauss’s Law in Coulomb gauge, this equation of motion is saturated by unphysical states only, whether the model confines or not. To see that all states that contribute to Eq. (95) are unphysical note that physical states $\vert \Psi _\mathrm{phys} \rangle$ are created by physical operators defined in Eq. (17). They have vanishing ghost number and are annihilated by the “Gauss-BRST” charge of Eq. (93),

96 $$\begin{aligned} Q_\mathrm{BRST} \vert \Psi _\mathrm{phys} \rangle = 0. \end{aligned}$$

The ghost field $c$ does not annihilate $\vert \Psi _\mathrm{phys} \rangle$, since its only effect is to create a ghost. Equation (96) thus has to be ensured by gluonic contributions only, and one gets back Gauss’s law as the subsidiary condition,

97 $$\begin{aligned} \frac{\delta S_C}{\delta A_0^a(x)}\, \vert \Psi _\mathrm{phys} \rangle = 0 \qquad \forall \,x . \end{aligned}$$

Any non-vanishing contribution to Eq. (95) thus must be due to unphysical $\vert \psi \rangle \not \in \{\vert \Psi _\mathrm{phys} \rangle \}$. In Appendix C we give an explicit calculation of the r.h.s. of Eq. (95), and we relate the propagator of the temporal gluon to the Faddeev–Popov operator to show that it is saturated by instantaneous contributions only.

The discussion of the spatial components of the gluonic QEoM is very similar to that in LCG. The equation of motion for the spatial part of the gluon propagator is given by

98 $$\begin{aligned} \delta ^{ab}\delta _{ij}&= \left\langle A_j^b(y)\,\frac{\delta S_C}{\delta A_i^a(x)} \right\rangle _\mathcal {FT}\nonumber \\&= -\left\langle A_j^b(y)\, \left( \partial _\nu F_{\nu i}^a(x) + j^a_i(x)\right) \right\rangle _\mathcal {FT}\nonumber \\&+ i \left\langle A_j^b(y)\,s(D_i\bar{c}) ^a \right\rangle _\mathcal {FT}. \end{aligned}$$

The first matrix element necessarily is spatially transverse in Coulomb gauge with $\partial _i A_i=0$. The Faddeev–Popov operator of Coulomb gauge is instantaneous,

99 $$\begin{aligned} M^{ab}(x,y)&=- \partial _iD_i^{ab}\delta (\mathbf y - \mathbf x ) \delta (y_0-x_0)\end{aligned}$$

100 $$\begin{aligned}&:= \delta (y_0-x_0) M^{ab}(\mathbf x ,\mathbf y ), \end{aligned}$$

and the contribution of the last term in Eq. (98) therefore is instantaneous,

101 $$\begin{aligned} i \left\langle A_j^b(y)\,s\bar{s} A_i^a \right\rangle&= -i \left\langle (D_j c)^b(y)\,(D_i\bar{c})^a(x) \right\rangle \nonumber \\&= -i \left\langle (D_j^{bc}(y) \,(D_i^{ad}(x)\left[ M^{-1}(\mathbf{y, x }) \right] ^{cd} \right\rangle \nonumber \\&\delta (y_0-x_0). \end{aligned}$$

Its Fourier transform depends on spatial momenta only. The QEoM of the ghost gives the longitudinal part of the correlation function

102 $$\begin{aligned} -i \left\langle (D_j c)^b(y)\,(D_i\bar{c})^a(x) \right\rangle _\mathcal {FT}= - t_{ij} u_C(\mathbf p ^2) + l_{ij}, \end{aligned}$$

where $t_{ij}$ and $l_{ij}$ are the longitudinal and transverse spatial projectors. The confinement criterion in Coulomb gauge reads

103 $$\begin{aligned} \lim _\mathbf{p ^2\rightarrow 0} u_C(\mathbf p ^2) = -1 \qquad \text { and} \qquad \lim _\mathbf{p ^2\rightarrow 0} f_C(p_0,\mathbf p ) = 0, \end{aligned}$$

where the function $f_C(p_0,\mathbf p )$ is defined by

104 $$\begin{aligned} -\left\langle A_k^b(y)\,\partial _\nu F_{\nu j}^a(x) \right\rangle _\mathcal {FT}= \delta ^{ab}t_{kj} f_C(p_0,\mathbf p )\, . \end{aligned}$$

The conditions of Eq. (103) ensure that the spatial gluonic QEoM is saturated by unphysical degrees of freedom in Coulomb gauge.

It is interesting that the correlation function in Eq. (101) is related to the horizon function of minimal Coulomb gauge in a finite quantization volume $V$ [56–59],

105 $$\begin{aligned} H(A)&\equiv -i \delta ^{ab}\delta _{ij} \int \!\hbox {d}^3x\hbox {d}^3y \, \nonumber \\&\quad \times (D_j^{bc}(y) \, (D_i^{ad}(x)\left[ M^{cd}(\mathbf{y, x }) \right] ^{-1}, \end{aligned}$$

with

106 $$\begin{aligned} \left\langle H(A) \right\rangle = V (N_c^2 - 1) \lim _\mathbf{p ^2\rightarrow 0}(1-2 u_C(\mathbf p ^2)) . \end{aligned}$$

In minimal Coulomb gauge, the configuration space is constrained to the first Gribov region by imposing the constraint

107 $$\begin{aligned} \left\langle H(A) \right\rangle = 3 V (N_c^2 - 1), \end{aligned}$$

which in fact is equivalent to the condition $u_C(0) = -1$ for color confinement of Eq. (106). A similar relation between the Kugo–Ojima criterion and the horizon condition also holds in minimal Landau gauge [58, 65], to which we now turn.

### Saturation and confinement in the covariant Gribov–Zwanziger(GZ) theory 

To avoid summation over gauge equivalent configurations, the GZ approach seeks to dynamically restrict the path integral to the first Gribov region [55–59]. The restriction leads to a horizon condition similar to Eq. (107) and can be implemented in a local renormalizable field theory with additional auxiliary ghosts. It was shown [59, 60] that the GZ Lagrangian differs from $\mathcal L_\mathrm{YM}$ by BRST-exact terms only. An infrared analysis of the GZ action reveals that its scaling solution coincides exactly with the solution calculated from the Faddeev–Popov action for Landau gauge [62, 63]: the ghost propagator is infrared enhanced and the gluon propagator infrared suppressed, the respective infrared exponents are identical. This corroborates the argument that for functional equations it suffices to take into account the appropriate boundary conditions, and no explicit restriction in the path-integral measure is required. However, the horizon condition implies that the BRST symmetry of the GZ action is spontaneously broken. In this last section we want to investigate how the gluonic QEoM in minimal Landau gauge is saturated in the infrared, even though the spontaneously broken BRST symmetry prohibits a definition of physical operators as in the foregoing sections.

The auxiliary ghost-fields, $\phi ^{a}_{\mu b},\bar{\phi }^{a}_{\mu b},\omega ^{a}_{\mu b}, \text {and } \bar{\omega }^{a}_{\mu b}$ are vector fields with two color indices that transform under the adjoint representation of the global color group (in $\hbox {SU}(3)$ they are reducible $\mathbf {8}\otimes \mathbf {8}=\mathbf {1}\oplus \mathbf {8}\oplus \mathbf {27} \oplus \mathbf {8}\oplus \mathbf {10}\oplus \overline{\mathbf {10}}$ color tensors),

108 $$\begin{aligned} \delta \Psi ^{a}_{\mu b} = g f^{acd} \Psi _{\mu b}^{c} \delta \vartheta ^d + g f^{bcd}\Psi _{\mu c}^{a}\delta \vartheta ^d \end{aligned}$$

for any $\Psi ^{a}_{\mu b} \in \{\phi ^{a}_{\mu b},\bar{\phi }^{a}_{\mu b},\omega ^{a}_{\mu b}, \bar{\omega }^{a}_{\mu b}\}$. While $\phi ^{a}_{\mu b}$ and $\bar{\phi }^{a}_{\mu b}$ are bosonic, $\omega ^{a}_{\mu b}$ and $\bar{\omega }^{a}_{\mu b}$ are fermionic. The auxiliary ghosts form a BRST quartet,

109 $$\begin{aligned} s\phi ^{a}_{\mu b}&= \omega ^{a}_{\mu b}, \quad s\omega ^{a}_{\mu b} = 0, \end{aligned}$$

110 $$\begin{aligned} s\bar{\omega }^{a}_{\mu b}&= \bar{\phi }^{a}_{\mu b}, \quad s\bar{\phi }^{a}_{\mu b} = 0 . \end{aligned}$$

Including this auxiliary quartet in the BRST transformations of Eq. (46), the BRST-exact part of the GZ Lagrangian is

111 $$\begin{aligned} \mathcal L_\mathrm{GZ}^{gf} = s(i\partial _\mu \bar{c}^a A^a_\mu + (\partial _\mu \bar{\omega }^{a}_{\nu b}) D_\mu ^{ac}\phi _{\nu b}^{c} ). \end{aligned}$$

The restriction of configuration space to the first Gribov region can be interpreted as a spontaneous breakdown of this BRST symmetry. As in any instance of a spontaneously broken symmetry it is advantageous to express the Lagrangian in terms of fluctuations about the symmetry breaking ground state. In the GZ framework this amounts to a shift of the fields by

112a $$\begin{aligned} \phi ^{a}_{\mu b}(x)&= \varphi ^{a}_{\mu b}(x) - \gamma ^{1/2} x_\mu \delta ^{a}_b ,\end{aligned}$$

112b $$\begin{aligned} \bar{\phi }^{a}_{\mu b}(x)&= \bar{\varphi }^{a}_{\mu b}(x) + \gamma ^{1/2} x_\mu \delta ^{a}_b , \end{aligned}$$

112c $$\begin{aligned} b^a(x)&= b^a(x) + i \gamma ^{1/2}x_\mu \hbox {tr}^{a}\!\left\{ \bar{\varphi }_\mu \right\} (x),\end{aligned}$$

112d $$\begin{aligned} \bar{c}^a(x)&= \bar{c}^a(x) + i \gamma ^{1/2}x_\mu \hbox {tr}^{a}\!\left\{ \bar{\omega }_\mu \right\} (x), \end{aligned}$$

where $\hbox {tr}^{a}\!\left\{ \Psi _\mu \right\} = g f^{abc} \Psi ^{b}_{\mu c}$.^2^ This change of variables in Eq. (111) gives the GZ Lagrangian of minimal Landau gauge [59, 60],

113 $$\begin{aligned} \mathcal L_\mathrm{GZ}^{gf}&= s(i\partial _\mu \bar{c}^a A^a_\mu +(\partial _\mu \bar{\omega }^{a}_{\nu b}) D_\mu ^{ac}\varphi _{\nu b}^{c} -\gamma ^{1/2} D_\mu ^{ac} \bar{\omega }^{c}_{\mu a} ) \nonumber \\&= i \partial _\mu b^a A_\mu ^a - i (\partial _\mu \bar{c}^a)(D_\mu c)^a +(\partial _\mu \bar{\varphi }^{a}_{\nu b})D_\mu ^{ac} \varphi _{\nu b}^{c} \nonumber \\&\quad - (\partial _\mu \bar{\omega }^{a}_{\nu b})D_\mu ^{ac}\omega _{\nu b}^{c}-(\partial _\mu \bar{\omega }^{a}_{\nu b}) \left( D_\mu c\! \times \! \varphi _{\nu b} \right) ^{a} \nonumber \\&\quad + \gamma ^{1/2} \left( D_\mu ^{ac} (\varphi ^{c}_{\mu a} - \bar{\varphi }^{c}_{\mu a}) -\left( D_\mu c\! \times \! \bar{\omega }_{\mu a} \right) ^{a} \right) - \gamma d N_c. \end{aligned}$$

Although the shift (112) and the BRST transformations are $x$-dependent, the shifted Lagrangian (113) does not include any explicit $x$-dependence, and it is Poincaré invariant.

The BRST variations of the shifted quantum fields are^3^

114a $$\begin{aligned} sA_\mu ^a&= (D_\mu c)^a, \quad sc^a=-\frac{1}{2} \left( c\! \times \! c \right) ^{a} ,\end{aligned}$$

114b $$\begin{aligned} s\bar{c}^a&= b^a, \quad sb^a = 0 , \end{aligned}$$

114c $$\begin{aligned} s\varphi ^{ab}_\mu&= \omega ^{ab}_\mu , \quad s\omega ^{a}_{\mu b} = 0 , \end{aligned}$$

114d $$\begin{aligned} s \bar{\omega } ^{ab}_\mu (x)&= \bar{\varphi }^{ab}_\mu (x) + \gamma ^{1/2}\delta ^{ab}x_\mu , \quad s\bar{\varphi }^{ab}_\mu = 0 . \end{aligned}$$

The Gribov parameter $\gamma$ is found by demanding that the model is quantized about an extremum of the quantum effective action $\Gamma$,

115 $$\begin{aligned} \frac{\delta \Gamma }{\delta \gamma } = 0. \end{aligned}$$

The inhomogeneous term of Eq. (114d) causes the BRST symmetry of the local Lagrangian to be spontaneously broken for any extremum of the quantum action with non-vanishing $\gamma$. It is perhaps worth noting that for $\gamma \ne 0$ the Poincaré generators do not commute with the BRST charge even though Poincaré invariance is *not* spontaneously broken.

To proceed with our investigation of confinement criteria in various gauges, we consider the gluonic QEoM implied by the Lagrangian $\mathcal L_\mathrm{GZ} = \mathcal L_\mathrm{YM}+ \mathcal L_\mathrm{GZ}^{gf}$

116 $$\begin{aligned} \frac{\delta S_\mathrm{GZ}}{\delta A_\mu ^a}&= - \partial _\nu F_{\nu \mu }^a -j_\mu ^{LCG\,a} + s(D_\mu \bar{c})^a\nonumber \\&\quad + \left( \varphi \! \times \! \partial _\mu \bar{\varphi } \right) ^{a} + \left( \omega \! \times \! \partial _\mu \bar{\omega } \right) ^{a} - \left( c\! \times \! \left( \partial _\mu \omega \! \times \! \varphi \right) ^{} \right) ^{a} \nonumber \\&\quad - \gamma ^{1/2} \left( c\! \times \! \hbox {tr}^{}\!\left\{ \omega _\mu \right\} \right) ^{a} + \gamma ^{1/2}\hbox {tr}^{a}\!\left\{ \varphi _\mu -\bar{\varphi }_\mu \right\} , \end{aligned}$$

where we suppressed all indices that are summed. Contractions with structure constants in the “covariant” and “contravariant” color indices are denoted by

117 $$\begin{aligned} \left( \Psi \! \times \! \Omega \right) ^{a} = g f^{acd} \Psi ^{c}_{\mu b} \Omega ^{d}_{\mu b} \end{aligned}$$

and

118 $$\begin{aligned} \left( \Psi \,{\widetilde{\times }}\, \Omega \right) ^{a} = g f^{acd} \Psi ^{b}_{\mu c} \Omega ^{b}_{\mu d}. \end{aligned}$$

Equation (116) includes the global color current $j_\mu ^{\mathrm{LCG}\,a}$ of LCG given in Eq. (48). However, $j_\mu ^{\mathrm{LCG}\,a}$ is not the conserved color current of the GZ action since the auxiliary fields transform according to Eq. (108). The corresponding conserved color current is

119 $$\begin{aligned} j_\mu ^{\mathrm{GZ}\,a}&= j_\mu ^{\mathrm{LCG}\,a} + \left( c\! \times \! \left( \partial _\mu \bar{\omega }\! \times \! \varphi \right) ^{} \right) ^{a} +\gamma ^{1/2}\left( c\! \times \! \hbox {tr}^{}\!\left\{ \bar{\omega }_\mu \right\} \right) ^{a} \nonumber \\&\quad - \left( \varphi \! \times \! \partial _\mu \bar{\varphi } \right) ^{a} - \left( \varphi \,{\widetilde{\times }}\, \partial _\mu \bar{\varphi } \right) ^{a} -\left( \bar{\varphi }\! \times \! D_\mu \varphi \right) ^{a} \nonumber \\&\quad - \left( \bar{\varphi } \,{\widetilde{\times }}\, D_\mu \varphi \right) ^{a} - \left( \omega \! \times \! \partial _\mu \bar{\omega } \right) ^{a} -\left( \omega \,{\widetilde{\times }}\, \partial _\mu \bar{\omega } \right) ^{a} \nonumber \\&\quad + \left( \bar{\omega }\! \times \! D_\mu \omega \right) ^{a} + \left( \bar{\omega } \,{\widetilde{\times }}\, D_\mu \omega \right) ^{a} \nonumber \\&\quad + \left( \bar{\omega }\! \times \! \left( D_\mu c\! \times \! \varphi \right) ^{} \right) ^{a} +\left( \bar{\omega } \,{\widetilde{\times }}\, \left( D_\mu c\! \times \! \varphi \right) ^{} \right) ^{a} . \end{aligned}$$

Using Eq. (119) the QEoM of Eq. (116) may be rewritten

120 $$\begin{aligned} \frac{\delta S_\mathrm{GZ}}{\delta A_\mu ^a} = - \partial _\nu F_{\nu \mu }^a - j_\mu ^{\mathrm{GZ}\,a} + s\chi ^a_\mu , \end{aligned}$$

with

121 $$\begin{aligned} \chi ^a_\mu&= (D_\mu \bar{c})^a - \left( \bar{\omega }\! \times \! D_\mu \varphi \right) ^{a} -\left( \bar{\omega } \,{\widetilde{\times }}\, D_\mu \varphi \right) ^{a} \nonumber \\&- \left( \varphi \,{\widetilde{\times }}\, \partial _\mu \bar{\omega } \right) ^{a} - \gamma ^{1/2} \hbox {tr}^{a}\!\left\{ \bar{\omega }_\mu \right\} . \end{aligned}$$

The gluonic QEoM of the GZ action therefore has the now already familiar form

122 $$\begin{aligned} \delta ^{ab}\delta _{\mu \sigma }\delta (x-y)&= \left\langle A_{\sigma }^a(y)\,\frac{\delta S_\mathrm{GZ}}{\delta A_{\mu }^b(x)} \right\rangle \nonumber \\&= -\left\langle A_{\sigma }^a(y)\,(\partial _\nu F_{\nu \mu }^b + j_\mu ^{\mathrm{GZ}\,b})(x) \right\rangle \nonumber \\&+ i\left\langle A_{\sigma }^a(y)\,s\chi ^b_\mu (x) \right\rangle . \end{aligned}$$

Color transport is short ranged and the current matrix element does not contribute in the infrared if the functions $f_\mathrm{GZ}(p^2)$ and $u_\mathrm{GZ}(p^2)$ defined by

123a $$\begin{aligned} -\left\langle A_{\sigma }^a(y)\,\partial _\nu F_{\nu \mu }^b (x) \right\rangle _\mathcal {FT}&=T_{\sigma \mu } f_\mathrm{GZ}(p^2) \end{aligned}$$

123b $$\begin{aligned} i\left\langle A_{\sigma }^a(y)\,s(\chi ^b_\mu (x)) \right\rangle _\mathcal {FT}&=L_{\sigma \mu }-T_{\sigma \mu }u_\mathrm{GZ}(p^2), \end{aligned}$$

satisfy the criteria

124 $$\begin{aligned} f_\mathrm{GZ}(0)=0\qquad \text {and}\qquad u_\mathrm{GZ}(0)=-1. \end{aligned}$$

However, in this case of a spontaneously broken BRST symmetry it is not entirely clear that

125 $$\begin{aligned} 0&= \left\langle s(A_{\sigma }^a(y)\, \chi ^b_\mu (x)) \right\rangle \nonumber \\&= \left\langle A_{\sigma }^a(y)\, s(\chi ^b_\mu (x)) \right\rangle +\left\langle (D_\sigma c)^a(y)\, \chi ^b_\mu (x) \right\rangle \ \end{aligned}$$

holds, which would imply that only (unphysical) quartet states contribute to the matrix element of Eq. (123b). Due to the equations of motion of the antighost $\bar{c}$ and of the NL field $b^a$ the longitudinal part of Eq. (125) is satisfied. Although a proof is lacking, it therefore is at least plausible that the transverse part of Eq. (125) also holds.

The GZ action incorporates non-perturbative features and in fact satisfies the criteria (124) already at tree level. Expanding the gluonic QEoM (120) to tree level yields

126 $$\begin{aligned} \delta ^{ab}\delta _{\sigma \mu }&= \left\langle A_\sigma ^b(y) \frac{\delta S_\mathrm{GZ}}{\delta A_\mu ^a(x)} \right\rangle _{FT}\nonumber \\&\approx p^2 T_{\mu \nu } \left\langle A_\sigma ^b(y)\, A_\nu ^a(x) \right\rangle _{FT} + \left\langle A_\sigma ^b(y) \,i\partial _\mu b^a(x) \right\rangle _{FT} \nonumber \\&\quad + \gamma ^{1/2} g f^{acd} \left\langle A_\sigma ^b(y) \, (\varphi ^{c}_{\mu d} -\bar{\varphi }^{c}_{\mu d})(x) \right\rangle _{FT}. \end{aligned}$$

We again have that the longitudinal part of the gluon propagator is saturated by the NL field as in the foregoing investigations. The transverse part of Eq. (126) is satisfied by the tree-level propagators, given for example in [59, 87] (with $\lambda ^4 = 2 N_c g^2 \gamma$)

127 $$\begin{aligned} \left\langle A_\sigma ^b(y)\, A_\mu ^a(x) \right\rangle _{FT} \approx \delta ^{ab} \,T_{\sigma \mu } \,\frac{p^2}{p^4 + \lambda ^4} \end{aligned}$$

and,

128 $$\begin{aligned} \left\langle A_\sigma ^b(y) \, (\varphi ^{c,d}_\mu - \bar{\varphi }^{c,d}_\mu )(x) \right\rangle _{FT} \approx f^{bcd} \,T_{\sigma \nu }\, \frac{2g \gamma ^{1/2}}{p^4 + \lambda ^4} . \end{aligned}$$

The GZ gluon propagator vanishes in the infrared, and $f_\mathrm{GZ}(0)=0$. In addition the last term in Eq. (126), derived entirely from the BRST-exact term in Eq. (120), saturates the transverse part of the gluonic QEoM at tree level for vanishing momenta,

129 $$\begin{aligned} \delta ^{ab}T_{\sigma \mu } = \delta ^{ab}T_{\sigma \mu } \frac{p^4}{p^4+\lambda ^4} + \delta ^{ab}T_{\sigma \mu } \frac{\lambda ^4}{p^4+\lambda ^4} . \end{aligned}$$

Quite strikingly, both criteria of Eq. (124) for a confining phase are thus satisfied by the GZ Lagrangian already at tree level. Perturbative calculations to one- and two-loop order in 3 [88] and 4 [87, 89, 90] Euclidean dimensions as well as a non-perturbative infrared analysis [62, 63] of the GZ action show that in the infrared the gluon propagator remains suppressed and the ghost propagator diverges more strongly than a massless pole [58, 62, 63]. This infrared behavior agrees with the original Kugo–Ojima scenario [2].

## Conclusion

In summary, we have formulated as confinement criterion that the gluonic QEoM be saturated by unphysical states in the infrared. In the Higgs and Coulomb phases this is not the case. These conditions thus are sufficient for distinguishing a color-confining phase from a Higgs and a Coulomb phase in linear covariant (LCG), generalized linear covariant(GLCG), maximal Abelian (MAG), and Coulomb (CG) gauges. Although the details depend somewhat on the chosen gauge, a universal qualitative criterion emerges in theories with an unbroken BRST or equivariant BRST symmetry that distinguishes between physical and unphysical states.

In the considered gauges the QEoM of the gauge boson propagator is of the form

130 $$\begin{aligned} \delta _{\sigma \mu }\delta ^{ab} = -\left\langle A^a_\sigma (y)\, \partial _\nu F^b_{\nu \mu }(x) \right\rangle _\mathcal {FT}-\left\langle A^a_\sigma (y)\, \tilde{j}^b_\mu (x) \right\rangle _\mathcal {FT},\nonumber \\ \end{aligned}$$

where the local current $\tilde{j}_\mu ^a(x)$ differs from the canonical Noether current $j^b_\mu (x)$ of the model by a BRST-exact contribution only,

131 $$\begin{aligned} \tilde{j}_\mu ^a(x)=j_\mu ^a(x) +s \xi _\mu ^a(x). \end{aligned}$$

In models with unbroken BRST symmetry, $\tilde{j}_\mu ^a$ thus is physically equivalent to the conserved Noether current $j_\mu ^a$. The criteria distinguish the phases depending upon which term on the right-hand side of Eq. (130) saturates the unity on the left in the infrared.

For the models we considered, the generalized color current $\tilde{j}_\mu ^a(x)$ is given by

132a $$\begin{aligned} \tilde{j}_\mu (x)&={j_\mu }^{{U(1)}}-i\partial _\mu b&\qquad \text {linear covariant Abelian U(1) (Eq. (19))},\end{aligned}$$

132b $$\begin{aligned} \tilde{j}_\mu ^a(x)&=j^{LCG\,a}_\mu -i s (D_\mu \bar{c})^a&\qquad \text {in LCG (Eq. (56))},\end{aligned}$$

132c $$\begin{aligned} \tilde{j}_\mu ^a(x)&=j^{GLCG\, a}_\mu -i s_\alpha (D_\mu \bar{c})^a&\qquad \text {in GLCG (Eq. (62))},\end{aligned}$$

132d $$\begin{aligned} \tilde{j}_\mu (x)&=j^\mathrm{MAG}_\mu -i \partial _\mu b&\qquad \text {SU(2) in MAG (Eq. (79))},\end{aligned}$$

132e $$\begin{aligned} \tilde{j}_k^a(x)&=j^{C\, a}_k -i s (D_k \bar{c})^a&\qquad \text {spatial components in Coulomb gauge (Eq. (98))},\end{aligned}$$

132f $$\begin{aligned} \tilde{j}_\mu ^a(x)&=j^{\mathrm{GZ}\, a}_\mu -i s\chi _\mu ^a&\qquad \text {in GZ (Eq. (122))}. \end{aligned}$$

In all gauges with unbroken BRST or equivariant BRST symmetry $\tilde{j}_\mu (x)$ is physically equivalent to the conserved current $j_\mu (x)$ because the additional terms either are BRST-exact or vanish on the physical Hilbert space due to subsidiary conditions.

In the Coulomb phase, the first matrix element in Eq. (130), $f(p^2) \delta ^{ab}T_{\sigma \mu } =- \langle {A^a_\sigma (y)\, \partial _\nu F^b_{\nu \mu }(x)\rangle }_\mathcal {FT}$, does not vanish in the infrared. $f(0)\ne 0$ implies the existence of a massless vector boson.

In the Higgs and color-confining phases on the other hand, $f(0)=0$ and the current matrix element saturates Eq. (130) in the infrared limit $p^2\rightarrow 0$.

Since no gauge-invariant order parameter discriminates between the Higgs and color-confining phases [3], the question arises whether one can distinguish between them at all. All physical states are colorless in both phases [8, 91]. However, the states contributing to $\langle {A^a_\sigma (y)\, \tilde{j}^b_\mu (x)\rangle }_\mathcal {FT}$ at low momentum differ in these two phases. In the confining phase the infrared limit of the transverse part of this current matrix element is *entirely saturated by unphysical states*. One in particular can be sure that the phase is confining if a BRST-exact part of $\tilde{j}^a_\mu (x)$ saturates the current matrix element. In the Higgs phase physical states contribute to this matrix element.

We found that such criteria distinguishing the confining from the Higgs and Coulomb phases also exist for Abelian gauge theories. In the Abelian Coulomb phase, discussed in Sect. 2, the current does not saturate the QEoM of the photon propagator at low momenta and the photon is massless. At tree level in the Abelian Higgs model of Sect. 2.2, $f(0)\!=\!0$, and the massive physical vector boson saturates the transverse part of the current matrix element. The only BRST-exact contribution to the generalized current in this case is longitudinal. The SU(2) gauge theory in MAG, discussed in Sect. 3.3, can be viewed as an Abelian ${U(1)}$ gauge theory in a confining phase. If unphysical states created by the $[s_\varepsilon ,\bar{s}_\varepsilon ] A_\mu$-part of the Noether current saturate the current matrix element at vanishing momentum, the theory describes a confining phase. Only the mutually commuting color charges of the Cartan subgoup are conserved in non-Abelian gauge theories in MAG and an *unphysical* part of the corresponding Abelian *Noether current* can saturate the gluonic QEoM in the infrared.

The saturation in GLCG, considered in Sect. 3.2, resembles that in LCG originally discussed by Kugo and Ojima. In these gauges the model confines color if the BRST-exact term, $-i s_\alpha (D_\mu \bar{c})^a$, of $\tilde{j}_\mu ^a$ saturates the gluonic QEoM in the infrared.

In the non-Abelian Coulomb gauge studied in Sect. 3.4, only unphysical states that do not satisfy Gauss’s Law contribute to the temporal part of the gluonic QEoM in all phases (and at all momenta). The temporal part of the gluonic QEoM thus cannot discriminate between phases. However, the theory is again confining if the spatial part of the gluonic QEoM in Coulomb gauge is saturated by the BRST-exact $s(D_k \bar{c})^a$ term of $\tilde{j}^a_k$. The corresponding confinement criterion of the Coulomb gauge was in addition found to be identical to the horizon condition of minimal Coulomb gauge.

An equivalence between the Kugo–Ojima confinement criterion and the horizon condition of minimal Landau gauge has also been established in [58, 65]. The auxiliary fields also contribute to the conserved Noether currents $j_\mu ^{\mathrm{GZ}\,a}$ of the GZ action (see Eq. (119)), but the gluonic QEoM retains the form of Eq. (130) with $\tilde{j}_\mu ^a$ given by Eq. (132f). In this model the BRST-exact part $s\chi _\mu ^a$ of $\tilde{j}_\mu ^a$ saturates the gluonic QEoM at $p^2=0$ already at *tree level*. There is no massless vector boson, and the gluon propagator at low momentum is suppressed. The GZ-theory in this sense satisfies all the confinement criteria for gauge theories with BRST symmetry. However, at present it is not known how to define a physical Hilbert space in this model with a spontaneously broken BRST symmetry [59–61, 92] and one has to prove that the BRST-exact contribution of the generalized current does not create physical states.

## Appendix A: Notations and conventions

In this appendix we fix notations and conventions. Throughout this article gauge theories in four-dimensional Euclidean spacetime are considered.

For QED the covariant derivative of any field $\psi$ with electromagnetic charge $g$ is denoted by

133 $$\begin{aligned} D_\mu \psi = \partial _\mu \psi - i g A_\mu \psi \end{aligned}$$

where $A_\mu$ is the gauge connection. The corresponding Abelian field strength is $F_{\mu \nu } = \partial _\mu A_\nu - \partial _\nu A_\mu$, and the classical Maxwell Lagrangian is normalized such that $\mathcal L_A= \frac{1}{4} F_{\mu \nu }F_{\mu \nu }$.

The covariant derivative of a field in the adjoint representation of an $\hbox {SU}(N)$ Yang–Mills theory is written as

134 $$\begin{aligned} D_\mu ^{ab} \psi ^b = \partial _\mu \psi ^a + \left( A_\mu \! \times \! \psi \right) ^{a} \end{aligned}$$

where the cross product is given by the structure constants $f^{abc}$ of the group, $\left( \chi \! \times \! \psi \right) ^{a} = g f^{abc}\chi ^b \psi ^c$. In Sect. 3.3, we use the adjoint covariant derivative with respect to the gluon field in the Cartan subalgebra, defined by

135 $$\begin{aligned} \mathsf {D}^{ab}_\mu = \delta ^{ab}\partial _\mu + g f^{aib}A_\mu ^i. \end{aligned}$$

The non-Abelian field strength is defined by the relation

136 $$\begin{aligned} g F_{\mu \nu }^a = i \left[ D_\mu , D_\nu \right] ^a, \end{aligned}$$

and the classical Yang–Mills Lagrangian by $\mathcal L_\mathrm{YM}\!=\! \frac{1}{4}\,F^a_{\mu \nu }F_{\mu \nu }^a$.

The Fourier transform of a correlation function $\left\langle \mathcal {O}_1(y) \right\rangle$ ${ \mathcal {O}_2(x)}$ is defined as

137 $$\begin{aligned}&\left\langle \mathcal {O}_1(y)\,\mathcal {O}_2(x) \right\rangle _\mathcal {FT}\nonumber \\&\quad =\frac{1}{(2\pi )^4}\int \!\!\hbox {d}^4(y\!-\!x)\,\,e^{-ip_{\mu }(y-x)_\mu }\left\langle \mathcal {O}_1(y)\,\mathcal {O}_2(x) \right\rangle .\nonumber \\ \end{aligned}$$

We use an equivalent sign $\equiv$ between expressions that differ by terms that vanish when the classical equations of motion are satisfied and $\approx$ if expressions coincide to leading order, usually tree level.

## Appendix B: Proof of restored BRST symmetries in MAG

Here we prove that the expectation values of equivariant BRST and anti-BRST variations (given in Eq. (68)) of ${U(1)}$-invariant operators vanish for an $\hbox {SU}(2)$ gauge theory in MAG, cf. Eq. (76),

138 $$\begin{aligned} \left\langle \delta _x\mathcal {O} \right\rangle = \left\langle s_\varepsilon \mathcal {O} \right\rangle = \left\langle \bar{s}_\varepsilon \mathcal {O} \right\rangle = 0, \quad \text { for all } \mathcal {O}\in \mathfrak {W}, \end{aligned}$$

where Eq. (75) defines the space $\mathfrak {W}$ of ${U(1)}$-invariant operators. With mild restrictions on the topology of spacetime, i.e. the Laplace-operator has to have an inverse, Eq. (73a) for any $\mathcal {O}\in \mathfrak {W}$ implies that

139 $$\begin{aligned} \left\langle \mathcal {O}\ b(x) \right\rangle =0\ \quad \text {if}\ \mathcal {O}\in \mathfrak {W}. \end{aligned}$$

The variations of the ${U(1)}$-gauge field $A_\mu$ satisfy

140 $$\begin{aligned} \delta _xs_\varepsilon A_\mu =\delta _x\left( B_\mu \! \times \! c \right) ^{} =0 \end{aligned}$$

and

141 $$\begin{aligned} \delta _x \bar{s}_\varepsilon A_\mu =\delta _x \left( B_\mu \! \times \! \bar{c} \right) ^{} = 0. \end{aligned}$$

$s_\varepsilon A_\mu$ and $\bar{s}_\varepsilon A_\mu$ thus are local ${U(1)}$-invariant functionals although $A_\mu$ is not. Since the product of two ${U(1)}$-invariant operators is a ${U(1)}$-invariant operator, Eq. (139) implies that

142a $$\begin{aligned} 0 = \left\langle b(x)\ \mathcal {O}s_\varepsilon A_\mu (y) \right\rangle&= \left\langle \partial _\nu b(x)\,\mathcal {O}\, s_\varepsilon A_\mu (y)\ \right\rangle ,\end{aligned}$$

142b $$\begin{aligned} 0 = \left\langle b(x)\ \mathcal {O}\bar{s}_\varepsilon A_\mu (y) \right\rangle&= \left\langle \partial _\nu b(x)\,\mathcal {O}\, \bar{s}_\varepsilon A_\mu (y)\ \right\rangle . \end{aligned}$$

Contracting and taking the limit $y\rightarrow x$, Eq. (142) shows that the r.h.s. of Eqs. (73b) and (73c) vanish for functionals $\mathcal {O}\in \mathfrak {W}$. We thus have proven Eq. (138), that is, Eq. (76).

## Appendix C: The gluonic QEoM of the $A_0^a$ field

In this appendix we integrate out the $A_0$ field in Eq. (95) and show that the equation is saturated by instantaneous contributions only. With the action $\mathcal {S}_C$ given by the Lagrangian Eq. (91), the QEoM for the $A_0$-field is

143 $$\begin{aligned} \delta (x-y) \delta ^{ab}&= \left\langle A_0^b(y)\,\frac{\delta S_C}{\delta A_0^a(x)} \right\rangle \nonumber \\&= - \left\langle A_0^b(y)\,\left( D_i\left( D_i A_0 - \dot{A}_i\right) \right) ^a(x) \right\rangle . \end{aligned}$$

We decompose the right-hand side into two terms, so the gluonic QEoM reads

144 $$\begin{aligned} \delta (x-y) = I_1(x-y) + I_2(x-y), \end{aligned}$$

where

145 $$\begin{aligned} I_1(x-y)\delta ^{ab}&:= - \left\langle A_0^b(y)\, (D_i^2 A_0)^a(x) \right\rangle \nonumber \\ I_2(x-y) \delta ^{ab}&:= \left\langle A_0^b(y) \left( A_i\! \times \! \dot{A}_i \right) ^{a} \right\rangle . \end{aligned}$$

Here we used $(D_i \dot{A}_i)^a = \left( A_i\! \times \! \dot{A}_i \right) ^{a}$, which follows from the transverse Coulomb gauge condition $\partial _i A_i^a = 0$.

To improve readability in the following we suppress color indices and add them only where necessary. We wish to express these expectation values in terms of an integral over the canonical variables and make use of an identity proven in [93]:

146 $$\begin{aligned} \left\langle \mathcal {O}(A_i, A_0) \right\rangle&= \left\langle \mathcal {O}\left( A_i, i { \delta \over \delta \rho }\right) \exp \left( -i \int \hbox {d}^4x \ \rho A_0\right) \right\rangle \Big |_{\rho = 0}\nonumber \\&= N \int dE^\mathrm{tr} dA^\mathrm{tr} \ \mathcal {O}\left( A_i^\mathrm{tr}, i {\delta \over \delta \rho } \right) \nonumber \\&\quad \times \exp \int \hbox {d}^4x ( i E_i^\mathrm{tr} \dot{A}_i^\mathrm{tr} -\mathcal{H} ) \Big |_{ \rho = 0 }, \end{aligned}$$

where $\rho$ is a source for $A_0$. To obtain this formula, one introduces the color-electric field $E_i$ by an auxiliary integration, after which one integrates out $A_0$ and the longitudinal part of $E_i$. This takes one from the Faddeev–Popov formula for integrating over $\int \hbox {d}^4A = \int \hbox {d}^3A_i dA_0$ to an integration over the canonical variables of the Coulomb gauge, ${A^\mathrm{tr}}_i^b$ and ${E^\mathrm{tr}}_i^b$, which are the three-dimensionally transverse vector potential and chromoelectric field. In the last formula, the Hamiltonian density is given by

147 $$\begin{aligned} \mathcal{H} := \frac{1}{2} (E^2 + B^2), \end{aligned}$$

where

148 $$\begin{aligned}&B_i^a = \epsilon _{ijk} [\partial _j {A^\mathrm{tr}}_k^a +\frac{1}{2}f^{abc} {A^\mathrm{tr}}_j^b {A^\mathrm{tr}}_k^c],\nonumber \\&E_i = E_i^\mathrm{tr} - \partial _i \varphi , \\&\varphi = M^{-1}(\rho _\mathrm{coul} + \rho ),\nonumber \end{aligned}$$

and $M = - D_i(A^\mathrm{tr}) \partial _i$ is the Faddeev–Popov operator of Coulomb gauge. Here $\rho _\mathrm{coul} := - \left( A^\mathrm{tr}_i\! \times \! E^\mathrm{tr}_i \right) ^{}$ is the color-charge density of the dynamical degrees of freedom. If quarks were present we would have $\rho _\mathrm{coul}^a \equiv - \left( A^\mathrm{tr}_i\! \times \! E^\mathrm{tr}_i \right) ^{a} + g \bar{Q} \gamma _0 t^a q$. From identity (146) we obtain

149 $$\begin{aligned} \left\langle f(A_i) A_0(x) \right\rangle = \left\langle f(A_i) \ (-iK\rho _\mathrm{coul})(x) \right\rangle \end{aligned}$$

and

150 $$\begin{aligned}&\left\langle f(A_i) A_0(x) A_0(y) \right\rangle \nonumber \\&\quad = \left\langle f(A_i) \ [ \ K(x, y) - (K \rho _\mathrm{coul})(x) \ (K \rho _\mathrm{coul})(y) \ ] \right\rangle ,\nonumber \\ \end{aligned}$$

etc., where $(K\rho _\mathrm{coul})(x) \equiv \int \hbox {d}^4y \ K(x, y) \rho _\mathrm{coul}(y)$, and the color-Coulomb kernel is given by

151 $$\begin{aligned} K(x, y) \equiv [ M^{-1} ( - \partial _i^2) M^{-1}](x,y). \end{aligned}$$

The identity Eq. (150), when applied to Eq. (145), gives

152 $$\begin{aligned} I_1(x-y)&= \!-\! \left\langle \left[ D_i^2 K(x,y) \!-\! (D_i^2 K\rho _\mathrm{coul})(x) (K\rho _\mathrm{coul})(y) \!\right] \right\rangle \nonumber \\ \end{aligned}$$

153 $$\begin{aligned} I_2(x-y)&= \left\langle \left( A_i\! \times \! \dot{A}_i \right) ^{} \!\!(x) \, (-i)(K\rho _\mathrm{coul})(y) \right\rangle . \end{aligned}$$

We next separate the instantaneous and non-instantaneous parts of these expressions. The kernel $K(x,y) = K(\mathbf{x}, \mathbf{y}) \delta$ $(x_0 - y_0)$ is instantaneous, so the first term of $I_1$ is purely instantaneous. The second term of $I_1$ involves the canonical fields $E^\mathrm{tr}$ and $A^\mathrm{tr}$ at time $x_0$ and the canonical fields at time $y_0$. These are the dynamical degrees of freedom so their correlators are non-instantaneous.

Keeping only the instantaneous part in Eq. (152) one gets

154 $$\begin{aligned} I_1(x-y) = - \left\langle D_i^2 K(x,y) \right\rangle . \end{aligned}$$

To separate the instantaneous part in $I_2$, we shall express $\dot{A}_i^\mathrm{tr}$ in terms of the canonical fields $A_i^\mathrm{tr}$ and $E_i^\mathrm{tr}$. For this purpose we use the fact that the integral of a derivative vanishes,

155 $$\begin{aligned} 0&= \int dE^\mathrm{tr} dA^\mathrm{tr} \, \left( A^\mathrm{tr}_i(x)\! \times \! \frac{\delta }{\delta E^\mathrm{tr}_i(x)} \right) ^{a} \left[ \int \!\hbox {d}^4z \, K^{de}(y,z)\nonumber \right. \\&\left. \times \left( A^\mathrm{tr}_j(z)\! \times \! E^\mathrm{tr}_j(z) \right) ^{e} \ \exp \left( \int \! \hbox {d}^4x ( i E_i^\mathrm{tr}\dot{A}_i^\mathrm{tr} - \mathcal{H} )\right) \right] .\nonumber \\ \end{aligned}$$

Note that $\frac{\delta E_j^\mathrm{tr\,a}(z)}{\delta E_i^\mathrm{tr\,b}(x)} = \delta _{ij}^\mathrm{tr}(x-z) \delta ^{ab}$. Here $\delta _{ij}^\mathrm{tr}(x-z)$ is the kernel of the transverse projector $\delta _{ij}I - \partial _i (\partial ^2)^{-1} \partial _j$. This gives

156 $$\begin{aligned} 0&= \left\langle \, \left( A_i(x)\! \times \! [ i \dot{A}_i(x) - G_i(x)] \right) ^{a} \right. \nonumber \\&\times \,\int \!\hbox {d}^4z \ K^{de}(y,z) \left( A_j(z)\! \times \! E_j(z) \right) ^{e} \nonumber \\&\left. + g^2 f^{abc} f^{egc}A_i^b(x) \int \hbox {d}z \ K^{de}(y,z) A_j^g(z)\delta _{ij}^\mathrm{tr}(x-z ) \right\rangle ,\nonumber \\ \end{aligned}$$

where $G_i^c(x) \equiv { \delta \over \delta E_i^c(z)} \int \hbox {d}^4 z \ \mathcal{H}(z)$. The term in $G_i^c(x)$ involves dynamical fields $E^\mathrm{tr}$ and $A^\mathrm{tr}$ at time $x_0$ and the second factor involves these fields at time $y_0$ so the term in $G(x, y)$ is non-instantaneous. Keeping only the instantaneous parts, and, using $K^{de}(y, x) = K^{ed}(x, y)$, we obtain the identity

157 $$\begin{aligned}&\left\langle \left( A_i\! \times \! \dot{A}_i \right) ^{a} (x)\, (-i) (K\rho _\mathrm{coul})^d(y) \right\rangle \nonumber \\&\quad = \left\langle g^2 f^{abc} f^{cge} A_i^b(x) \int dz \ \delta _{ij}^\mathrm{tr} (x-z)\,A_j^g(z) \ K^{ed}(z, y) \right\rangle , \end{aligned}$$

where the left-hand side is $I_2$. Because of the transverse projector we may write this as

158 $$\begin{aligned} I_2 = \left\langle D_i \int \! \hbox {d}^4z \, \delta _{ij}^\mathrm{tr} (x-z) D_j K(z, y) \right\rangle . \end{aligned}$$

Equation (143) in operator notation now reads

159 $$\begin{aligned} \delta (x-y)&= \left\langle - D_i^2 K(x, y) +D_i \ \delta _{ij}^\mathrm{tr} D_j K(x, y)] \right\rangle \nonumber \\&= \left\langle \ - D_i \ \delta _{ij}^\mathrm{lo} D_j K(x, y)] \right\rangle \nonumber \\&= \left\langle - D_i \partial _i (\partial ^2)^{-1} \partial _j D_j K(x, y)] \right\rangle \nonumber \\&= \left\langle M (-\partial ^2)^{-1} M K(x, y)] \right\rangle , \end{aligned}$$

where $\delta _{ij}^\mathrm{lo} = \partial _i (\partial ^2)^{-1}\partial _j$, and so, with $K = M^{-1} (-\partial ^2) M^{-1}$, we obtain the identity

160 $$\begin{aligned} \delta (x-y) = \delta (x-y). \end{aligned}$$

We see that once the $A_0$-field has been integrated out, the gluonic QEoM is satisfied identically by the instantaneous parts only.

## Footnotes

1 Note that the definition of the function $u_\mathrm{MAG}(p^2)$ in MAG apparently differs by a factor of $(-2)$ from that of GLCG given by Eq. (63), since $(D_\mu c)^3= \partial _\mu c^3+\left( B_\mu \! \times \! c \right) ^{3}$ formally differs from $\left( B_\mu \! \times \! c \right) ^{}$ in Eq. (90) by a longitudinal contribution only. However, Eqs. (63) and (90) are gauge dependent correlation functions that are not required to coincide in two different gauges. In addition, as LCG and MAG are not analytically connected, [53], no quantitative relation should be expected between the functions $u_\mathrm{LCG}$ and $u_\mathrm{MAG}$.

2 Note that on a finite torus with *antiperiodic* boundary conditions for the auxiliary ghosts, this $x$-dependent shift can be interpreted as quantization about a classical solution to the equations of motion.

3 This global transformation may appear to go outside the framework of standard quantum field theory because of the large change at infinity. However, Noether’s theorem and the Ward identities based on it rely for their validity on the infinitesimal local transformation $s_\epsilon = \epsilon (x) s$, which acts in particular on $\bar{\omega } ^{ab}_\mu$ according to

$$\begin{aligned} s_\epsilon \bar{\omega } ^{ab}_\mu (x) = \epsilon (x)\bar{\varphi }^{ab}_\mu (x) + \gamma ^{1/2}\delta ^{ab} x_\mu \epsilon (x). \end{aligned}$$

Here $\epsilon (x)$ may be chosen to be zero outside a small but arbitrary region, so the transformation at large $x$ is strictly zero. It is sufficient that the variation of the local Lagrangian under this infinitesimal and local transformation be proportional to $\partial _\mu \epsilon$, which it is, $s_\epsilon \mathcal{L}_\mathrm{GZ} \sim j_\mu \partial _\mu \epsilon$. The global transformation may be sidestepped [59].
